# Supplementary material for: Direct Reprogramming of Murine Fibroblasts to Hematopoietic Progenitor Cells
Source: Cell Rep. 2014 Nov 26;9(5):1871–84. doi: 10.1016/j.celrep.2014.11.002 (PMC4542300; doi:10.1016/j.celrep.2014.11.002)
Supplement: Document S1. Supplemental Experimental Procedures, Figures S1–S6, and Tables S1 and S2 [file mmc1.pdf]

Cell Reports, Volume *9*

Supplemental Information

Direct Reprogramming of Murine Fibroblasts to Hematopoietic Progenitor Cells

Kiran Batta, Magdalena Florkowska, Valerie Kouskoff, and Georges Lacaud

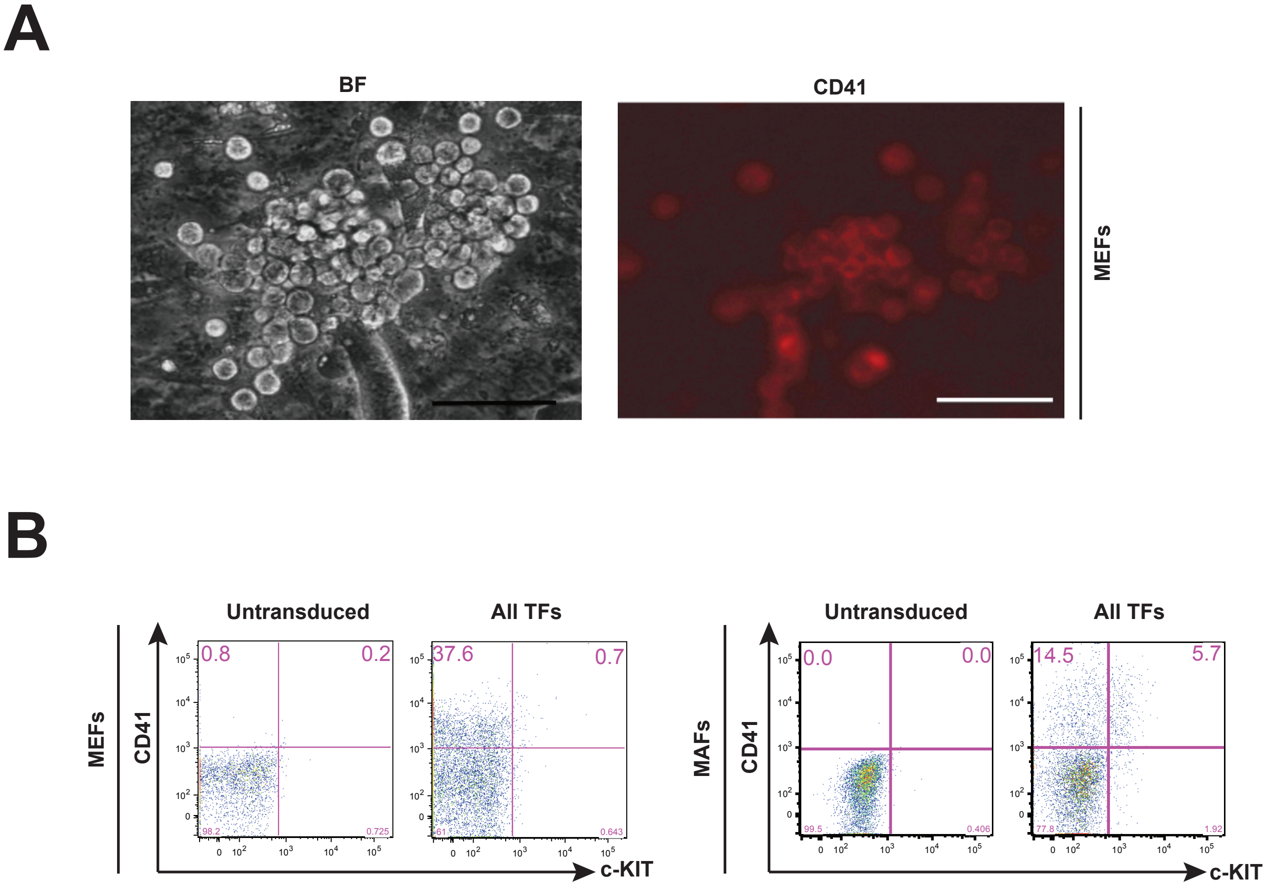

Figure S1 (related to Figure 1)

(A) Immunostaining of transduced MEFs at day 12 with a CD41-PE antibody. No staining was observed in untransduced MEFs. (B) FACS analyses for hematopoietic cell surface markers on all factor transduced MEFs and MAFs at day 21.

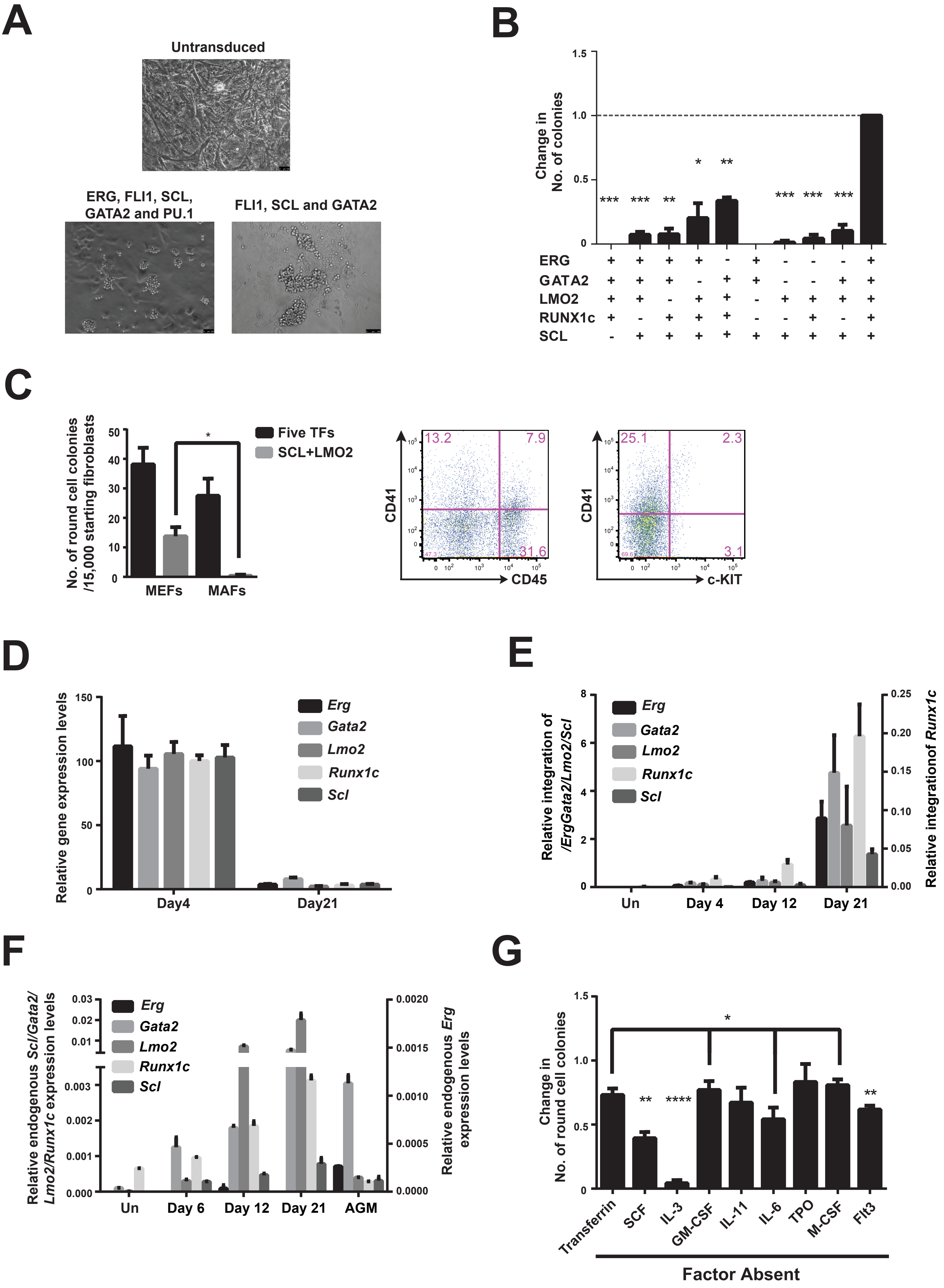
A
Untransduced
ERG, FLI1, SCL, GATA2 and PU.1
FLI1, SCL and GATA2
B
Change in No. of colonies
1.5
1.0
0.5
0
*** *** ** * ** *** *** ***
ERG + + + + - + - - - +
GATA2 + + + - + + - - + +
LMO2 + + - + + - + + + +
RUNX1c + - + + + - - + - +
SCL - + + + + + + + + +
C
No. of round cell colonies /15,000 starting fibroblasts
50
40
30
20
10
0
*
MEFs
MAFs
Five TFs
SCL+LMO2
13.2
7.9
47.3
31.6
CD41
CD45
25.1
2.3
69.6
3.1
CD41
c-KIT
D
Relative gene expression levels
150
100
50
0
Day4
Day21
Erg
Gata2
Lmo2
Runx1c
Scl
E
Relative integration of /ErgGata2/Lmo2/Scl
8
6
4
2
0
Relative integrationof Runx1c
0.25
0.20
0.15
0.10
0.05
0.00
Un
Day 4
Day 12
Day 21
Erg
Gata2
Lmo2
Runx1c
Scl
F
Relative endogenous Scl/Gata2/ Lmo2/Runx1c expression levels
0.03
0.02
0.01
0.003
0.002
0.001
0.000
Relative endogenous Erg expression levels
0.0020
0.0015
0.0010
0.0005
0.0000
Un
Day 6
Day 12
Day 21
AGM
Erg
Gata2
Lmo2
Runx1c
Scl
G
Change in No. of round cell colonies
1.5
1.0
0.5
0
*
** **** **
Transferrin
SCF
IL-3
GM-CSF
IL-11
IL-6
TPO
M-CSF
Flt3
Factor Absent

Figure S2 (related to Figure 2)

(A) Bright-field images of round cell colonies generated by transduced MEFs with the indicated TFs at day 18.

(B) Relative change in the number of round cell colonies visible at day 15 following transduction of MAFs with the indicated combination of TFs. The number of colonies generated by the complete five TFs combination is attributed a value of 1. Mean ± SEM of three independent experiments is shown (n=3) (C) Number of round cell colonies generated by 15K MEFs/MAFs transduced with either five TFs or only SCL and LMO2 (left). Mean ± SEM of two independent experiments is shown (n=2). FACS analysis of day 21 SCL and LMO2 transduced MEFs cultures (right). (D) Relative expression levels with respect to β-actin of exogenously introduced TFs in day 21 transduced cells and day 4 transduced cells (n=3, mean ± SD). (E) Relative integration of vectors encoding the different TFs in untransduced (Un) and transduced MEFs at day 4, day 12 and day 21 in respect to the endogenous *PARP* gene (n=3, mean ± SD). (F) Relative expression levels of endogenous genes with respect to β-*actin* in untransduced (Un) MEFs, day 6, day 12 and day 21 transduced MEFs and control E10.5 AGM cells. Mean ± SD of triplicates of a single experiment is shown. (G) Change in the number of round cell colonies generated after five TF transduction in media depleted of the indicated cytokines or growth factors. Mean ± SEM of four independent experiments is shown (n=4). Asterisk(s) indicates significant differences (Student t-test, *p <0.05, **p<0.01, ***p<0.0005, ****p<0.0001).

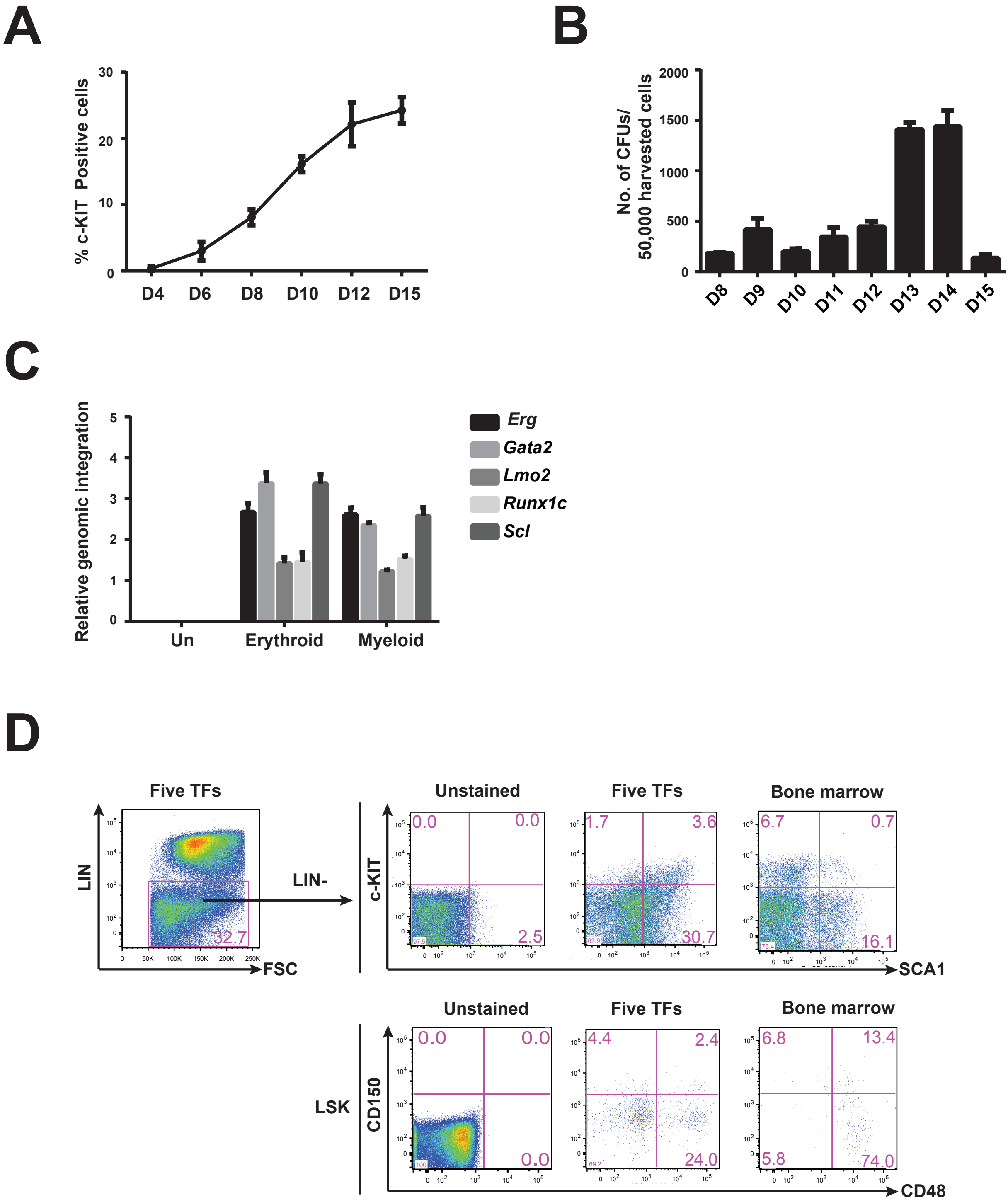

Figure S3 (related to Figure 3)

(A) FACS analyses of the emergence of c-KIT+ cells in cultures of transduced MAFs at the indicated days. Mean ± SEM of three independent experiments is shown (n=3) (B) CFU potential of five TF transduced MAFs at the indicated days of cultures. Bars represent mean ± SEM of triplicates of a representative experiment (n=3) (C) Relative genomic integration of vectors for all five TFs in untransduced (Un) and reprogrammed MEFs in respect to the endogenous *PARP* gene. MEFs transduced with five TFs were cultured on OP9. On day 14 cells were sorted into erythroid (TER119+) or myeloid (CD11b+) fractions and subjected to genomic DNA extractions and subsequent qRT-PCR. Data presented are mean ± SD of a representative experiment performed in triplicates (n=2). (D) FACS analyses using the indicated markers of phenotypic HSCs in reprogrammed MEFs expanded on OP9-DL1 for two weeks and control bone marrow.

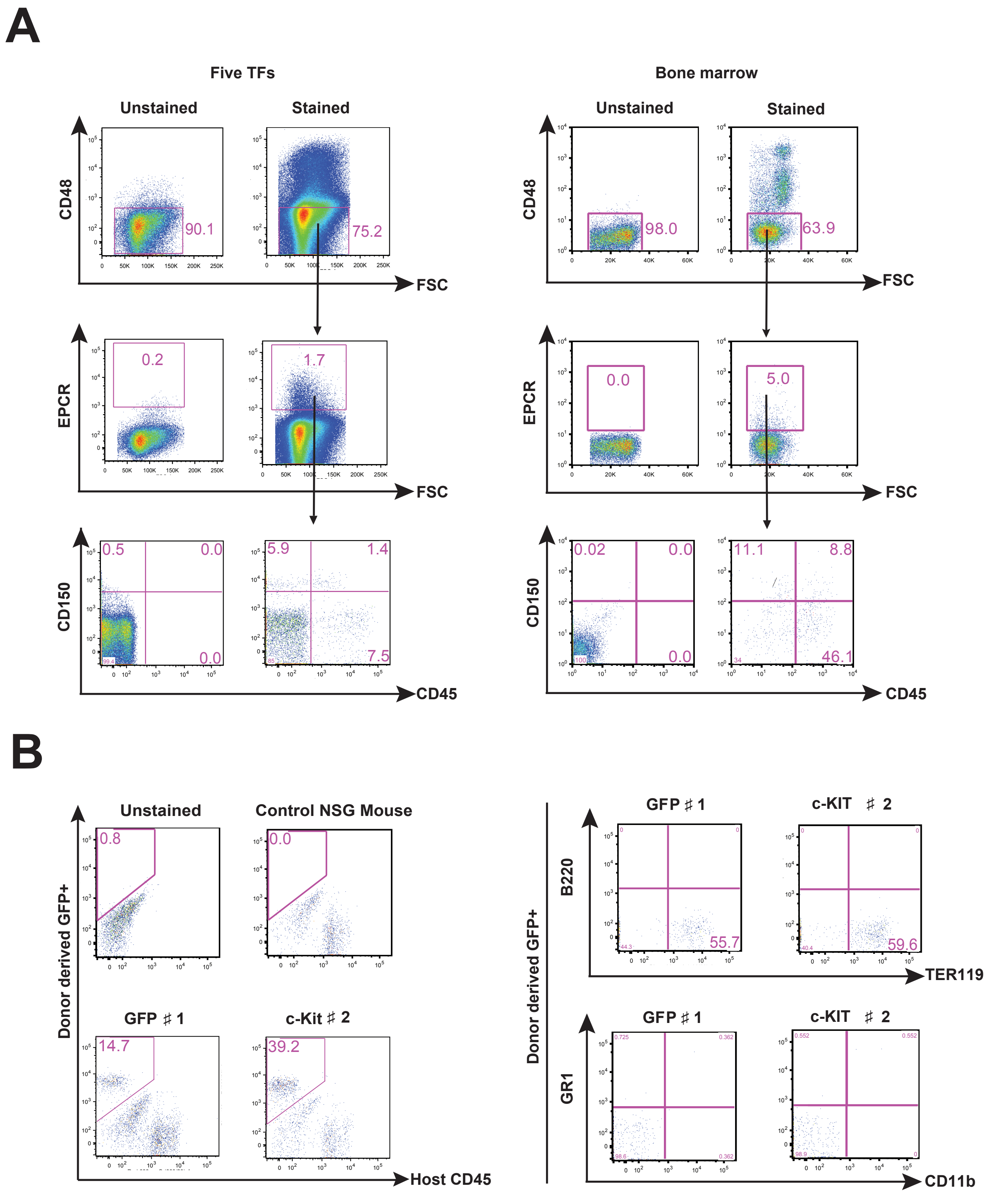

Figure S4 (related to Figure 3)

(A) FACS analyses using the indicated markers of phenotypic HSCs in reprogrammed MEFs expanded on OP9-DL1 for two weeks and control bone marrow. (B) Representative FACS analyses of peripheral blood of mice 2 weeks after transplantation for donor derived GFP positive cells and host CD45 (right). FACS analyses of donor derived cells (GFP gated) for the hematopoietic cell surface markers B220, TER119, GR1 and CD11b (left).

A

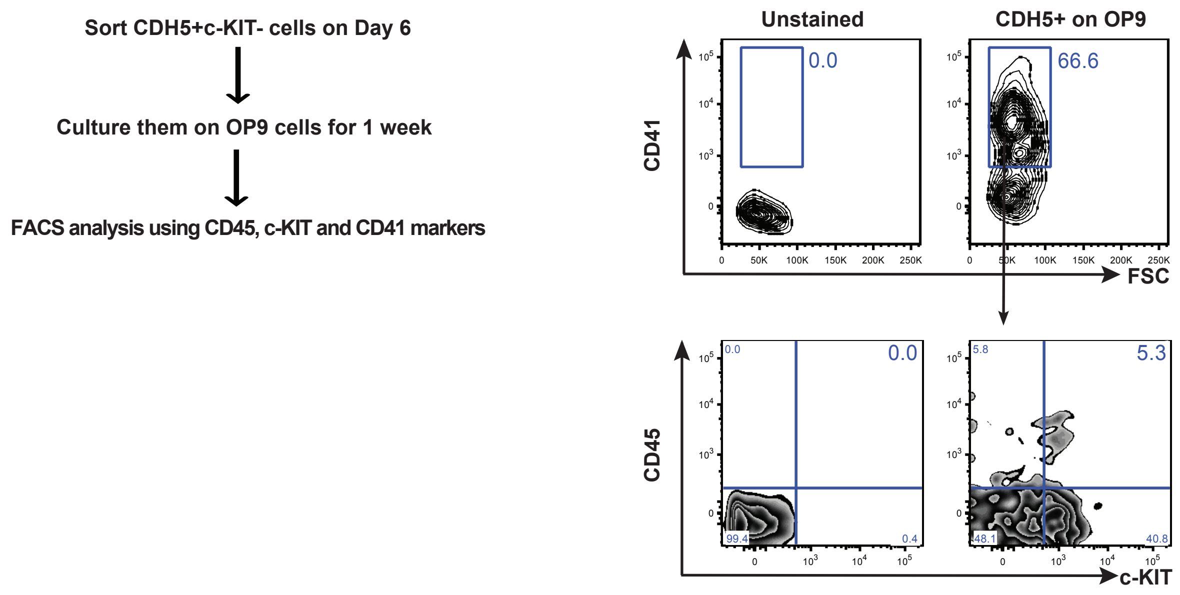

Figure S5 (related to Figure 5)

(A) CDH5+ c-KIT- cells were sorted from transduced day 6 MEFs and cultured on OP9 stromal layers for 7 days. FACS analyses were performed for the indicated markers (n=2).

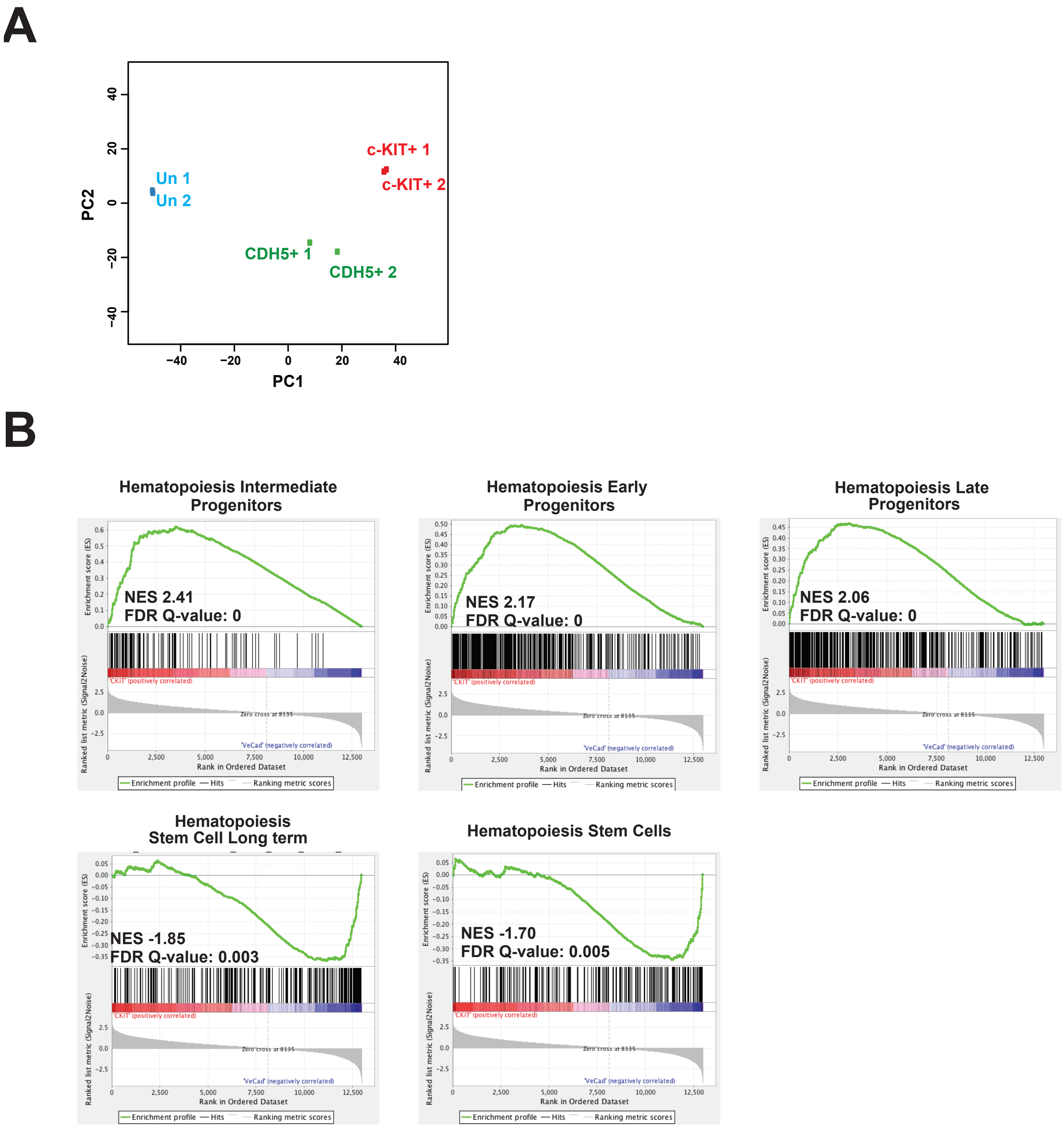

Figure S6 (realted to Figure 6)

(A) PCA analyses of DEGs among untreated (Un), CDH5+ and c-KIT+ positive cells. (B) GSEA analyses with the indicated gene data sets (Ivanova, 2002) comparing CDH5+ and c-KIT+ transcriptomes.

Table S1: (related to Figure 1)
List of the 19 TFs screened for reprogramming to blood

Gene Name	Gene ID
CEBP-α	NM_007678.3
CEBP-β	NM_009883.3
c-Fos	NM_010234.2
Erg	NM_133659.2
Ets1	NM_001038642.1
Etv2	NM_007959.2
Etv6	NM_007961.3
Fli1	NM_008026.4
GATA1	NM_008089.1
GATA2	NM_008090.4
Gfi1	NM_010278.2
Gfi1b	NM_008114.2
HoxB4	NM_010459.6
Lmo2	NM_008505.3
PU.1	NM_011355.1
Runx1b	NM_001111022.2
Runx1c	NM_001111023.2
Scl	NM_011527.2
Sox7	NM_011446.1

Table S2 : (related to Figure 6)

Gene ontology analysis of significantly enriched biological processes in the 3 clusters of genes differentially expressed among untreated fibroblasts, day 8 CDH5+ and day 12 c-Kit positive cells.

Cluster 1	Cluster 2	Cluster 3
Cell adhesion	Cell adhesion	Immune response
Biological adhesion	Biological adhesion	Defense response
Heart development	Angiogenesis	Leukocyte migration
Actin filament-based process	Blood vessel development	Cell activation
Extracellular matrix organization	Vasculature development	Response to wounding
Extracellular structure organization	Regulation of cell migration	Taxis
Enzyme linked receptor protein signalling pathway	Blood vessel morphogenesis	Leukocyte activation
Skeletal system development	Regulation of cell motion	Response to bacterium
Actin cytoskeleton organization	Regulation of locomotion	Mast cell activation

Movie S1: related to Figure 2

Time lapse imaging of 5-TFs transduced MEFs from day 3 to 13 of reprogramming.

Supplemental Experimental Procedures

TF cloning and lentiviruses production

Hematopoietic TFs cDNAs were cloned with a N-terminal FLAG tag in lentiviral vectors under the EF1 promoter. Viral particles were produced in HEK293T cells with third generation lentiviral packaging plasmids. Western blotting with FLAG antibodies was performed to quantitate and normalize the titers of viruses.

Flow cytometry

The following antibodies were used: CD45.1 APC (A20), CD45-PerCP Cy5.5 (30-F11;Biolegend), CD150-PerCP Cy5.5 (TC15-12F12.2), SCA1-PE-Cy7 (D7), CD201-APC (1560), CD48-PE (OX-78), CD48-APC (HM48-1), CD41-PE-Cy7 (MWReg30), c-KIT-APCeFluor780 (2B8), CDH5–APC (eBioBV13), TER119- PE-Cy7 (TER-119), CD11b-APC (M1/70), GR1-PE-Cy7 (RB6-8C5), CD19-PE-Cy7 (eBio1D3), B220-PE (RA3-6B2), CD25-APC (PC61.5), CD71-bio (R17217), CD31-Bio (Mec 13-3), CD41-Bio (eBio-MWReg30), CD45-Bio and PE-Cy7 (30-F11), c-KIT-Bio (2B8), Strep-BV421, Strep-PECy7 and mouse hematopoietic lineage biotin panel (all from eBioscience). Acquisitions were performed on LSRII (BD Biosciences) and data were analyzed using FlowJo software. Cell sortings were performed on AriaII or AriaIII sorters (BD Biosciences).

Lineage specific cultures

Multilineage potential of sorted c-KIT positive cells was tested by culturing them in (1X IMDM supplemented with plasma-derived serum (PDS;Antech), 10% protein-free hybridoma medium (PFM; Gibco), 0.5 mM Ascorbic Acid, 4.5x10-4 M MTG, 2 mM L-glutamine, 80 mg/ml transferrin and 50 µg/ml penicillin-streptomycin in addition to the following specific cytokines/growth factors; 1% IL3, 1% of GM-CSF and 10 ng/ml M-CSF for myeloid, 1% IL3, 1% c-KIT ligand and 4 U/ml Erythropoietin (Ortho-Biotech) for erythroid and 1% Thrombopoietin, 10 ng/ml IL6 and 5 ng/ml IL11 (R&D Systems) for megakaryocytes. Lymphoid cultures were carried out in the presence of 1% c-KIT ligand, 5 ng/ml Flt3 ligand and 1 ng/ml IL7 (B-lymphocytes) or 0.25 ng/ml (T-lymphocytes). To test presence of immunophenotypic HSCs day 12 c-KIT sorted five factor reprogrammed cells were cultured in medium supplemented with 1% c-KIT ligand, 1% IL3, 100 ng/ml Flt3 ligand and 10 ng/ml IL6 for two weeks.

Hematopoietic CFU-C assay

In order to evaluate the progenitor potential of the cells subjected to reprogramming, 50,000 cells were re-plated in triplicate in semi-solid media containing 55% methylcellulose (VWR) and supplemented with the cytokines used in reprogramming medium (see Viral transduction and culture). The cultures were maintained in

$37^0C/5\%$ CO_2 atmosphere and scored for the presence of hematopoietic colonies after 10-12 days.

Phagocytosis assay and cytospin analysis

Active phagocytosis was tested by adding 10 µl of fluorescent red conjugated carboxylate-modified polystyrene beads (0.5 um diameter, Sigma) to 100 µl aliquots of day 21 $CD45^+CD11b^+$ sorted reprogrammed cells ($3x10^6$/ml) and subsequent incubation for 2 hours at $37^0C/5\%$ CO_2 atmosphere with occasional shaking. Cells were washed twice with 10% FCS in PBS to remove any beads that were not phagocytised and subjected to standard cytospin procedure.
For cytospin analysis 30,000 cells suspended in 150 µl of medium were spun at 800 rpm for 5 minutes with low acceleration onto a glass slide. After air-drying cells were fixed in methanol for 10 minutes, washed and stained with May-Grunwald dye for 3 minutes. Following 5 rounds of washing, slides were submerged into Giemsa stain for 20 minutes, rinsed with water and air-dried. Subsequently mounted slides were scanned using Leica Scan Software.

Acetylcholinesterase staining

Acetycholinesterase staining was performed on cells generated from day 12 $c\text{-}KIT^+$ sorted reprogrammed $p53^{-/-}$ MEFs or whole bone marrow cells after culture in megakaryocytic medium. 30,000 cells were span onto glass slide at 800 rpm for 5 minutes and air dried. The cells were next fixed in ice-cold acetone for 5 minutes and air-dried. Samples were then covered with a staining solution of 0.5 mg/ml acetylthiocholine iodide, 5mM sodium citrate, 3mM copper(II) sulphate, 0.5mM potassium ferricyanide in 0.1M sodium phosphate buffer (pH6). Incubation was carried out for 3-6 hours in a humid chamber in the dark. The cells were then fixed in 95% EtOH for 10 minutes, rinsed with water and air dried. Counterstain in Harris' haematoxylin solution was carried out for 30 seconds immediately followed by brief destain in acid alcohol (1% HCl in 70% EtOH). Finally slides were rinsed with water and left to air dry. Images were captured on a Leica DMI3000B microscope.

Genomic PCR

Genomic DNA was isolated by lysis followed by proteinase K digestion. The integration of exogenous transcription factors was determined by PCR with SYBR green master mix and primers specific to FLAG tag and individual TF. For the detection of BCR and TCR gene rearrangements, $p53^{-/-}$ reprogrammed MEFs, cultured on OP9/OP9-DL1, were sorted for B220 and CD19 (B-lymphocytes) or CD25 (T-lymphocytes) respectively. Genomic DNA was isolated from 100.000 cells. D_H to J_H1-2 and D_H to J_H3 gene rearrangements were detected by nested PCR as reported earlier (Schlissel et al., 1991). TCR-β chain gene rearrangements were detected by a single round of PCR with the following primers: 5'-GCACCTGTGGGGAAGAAACT-3'; 5'-TGAGAGCTGTCTCCTACTATCGATT-3'. Products of both experiments were subjected to electrophoresis and the bands excised from the gel and sequenced.

Quantitative RT-PCR analysis

Total RNA was isolated using QIAGEN RNA Prep KIT (Qiagen) and cDNAs were generated with Omniscript RT kit (Qiagen). The QRT-PCR reactions were prepared using TaqMan Universal PCR Master Mix (Applied Biosystems) and universal probe library. The expression levels of endogenous TFs were determined with primers specific to the 3'UTR of their transcripts.

Immunofluorescence

For CDH5 staining, five TFs transduced cells cultured on gelatine-coated coverslips were washed with PBS and fixed with 0.4% formaldehyde (Sigma) in PBS for 20 minutes at RT. Cells were then permeabilized with a 0.2% TritonX (Sigma) solution in PBS before to be blocked with 10% goat serum (Gibco) for 10 minutes at RT. The cells were then incubated with a CDH5-biotin primary antibody (eBioBV14, eBioscience) at 1:100 dilution overnight at 4C and the next day with a secondary anti-rat Alexa647 antibody (Invitrogen) in 1:400 dilution for 1 hour. After washes, the cells were mounted with ProLong® Gold antifade reagent with DAPI (Invitrogen) and left at 4^0C overnight. Imaging was performed using a Zeiss Axiovert 200M microscope and a Andor iXon DU888+ camera. The system utilises Metamorph (Molecular Devices) software. Subsequent analysis was performed in Adobe Photsohop and ImageJ programmes.

Supplemental references

Ivanova, N.B. (2002). A stem cell molecular signature. Science *298*, 601–604.

Schlissel, M., Voronova, A., and Baltimore, D. (1991). Helix-loop-helix transcription factor E47 activates germ-line immunoglobulin heavy-chain gene transcription and rearrangement in a pre-T-cell line. Genes Dev *5*, 1367–1376.
