# Supplementary figures and images for: Direct Reprogramming of Murine Fibroblasts to Hematopoietic Progenitor Cells
Source: Cell Rep. 2014 Nov 26;9(5):1871–84. doi: 10.1016/j.celrep.2014.11.002 (PMC4542300; doi:10.1016/j.celrep.2014.11.002)

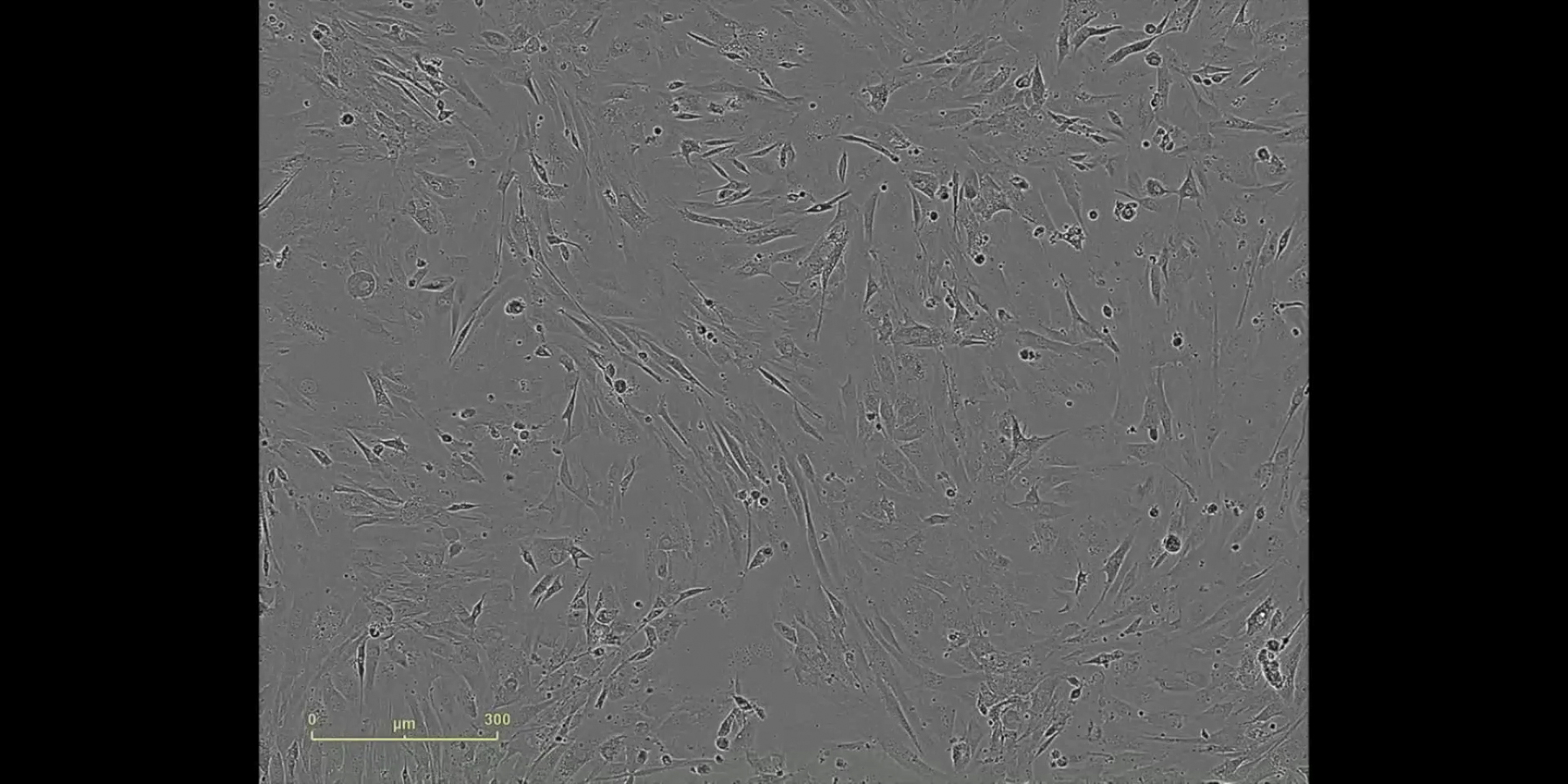

Supplement: Movie S1. Time-Lapse Imaging of Five-TF-Transduced MEFs from Day 3 to 13 of Reprogramming, Related to Figure 2 [file mmc2.jpg]
