# Supplementary material for: Direct Reprogramming of Murine Fibroblasts to Hematopoietic Progenitor Cells
Source: Cell Rep. 2014 Nov 26;9(5):1871–84. doi: 10.1016/j.celrep.2014.11.002 (PMC4542300; doi:10.1016/j.celrep.2014.11.002)
Supplement: Document S2. Article plus Supplemental Information [file mmc3.pdf]

Cell Reports

Article

Direct Reprogramming of Murine Fibroblasts to Hematopoietic Progenitor Cells

Graphical Abstract

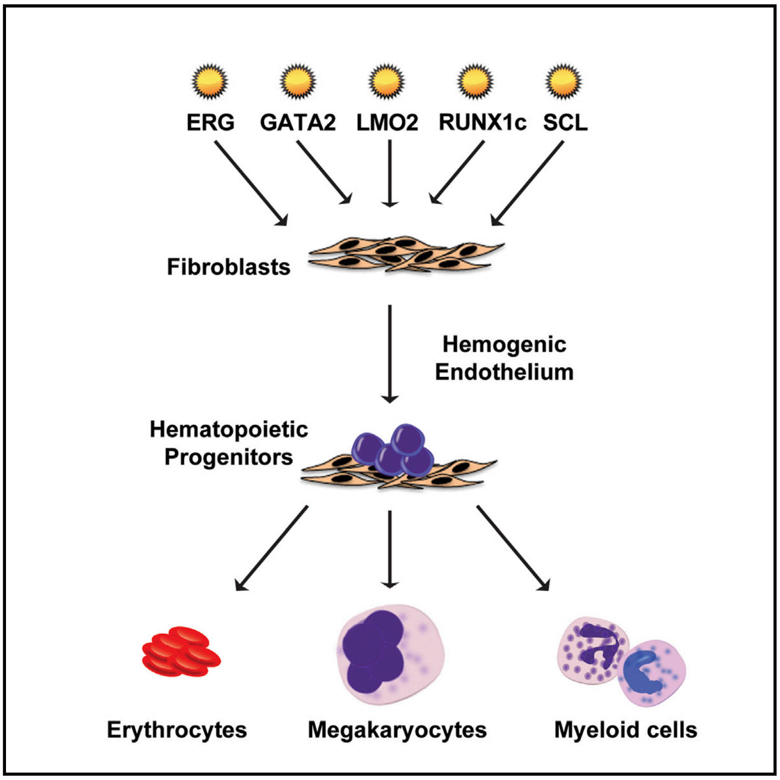

Highlights

ERG, GATA2, LMO2, RUNX1c, and SCL reprogram fibroblasts to blood

Reprogrammed fibroblasts have multilineage hematopoietic potential

Loss of p53 increases efficiency and multilineage potential of reprogrammed cells

Generation of blood progenitors is preceded by the appearance of hemogenic cells

Authors

Kiran Batta, Magdalena Florkowska, Valerie Kouskoff, Georges Lacaud

Correspondence

georges.lacaud@cruk.manchester.ac.uk

In Brief

Batta et al. demonstrate that murine fibroblasts are reprogrammed to hematopoietic progenitors, with erythroid, megakaryocyte, and myeloid potential, by ectopic expression of hematopoietic transcription factors. Reprogramming efficiency is increased by loss of p53 function, and generation of blood cells is preceded by the appearance of hemogenic endothelium.

Accession Numbers

GSE59428

Batta et al., 2014, Cell Reports 9, 1871–1884
December 11, 2014 ©2014 The Authors
http://dx.doi.org/10.1016/j.celrep.2014.11.002

OPEN ACCESS
CellPress

Direct Reprogramming of Murine Fibroblasts to Hematopoietic Progenitor Cells

Kiran Batta,[1,3] Magdalena Florkowska,[1,3] Valerie Kouskoff,[2] and Georges Lacaud[1,*]
[1]CRUK Stem Cell Biology Group, Cancer Research UK Manchester Institute, The University of Manchester, Wilmslow Road, Manchester M20 4BX, UK
[2]CRUK Stem Cell Haematopoiesis Group, Cancer Research UK Manchester Institute, The University of Manchester, Wilmslow Road, Manchester M20 4BX, UK
[3]Co-first author
*Correspondence: georges.lacaud@cruk.manchester.ac.uk
http://dx.doi.org/10.1016/j.celrep.2014.11.002
This is an open access article under the CC BY license (http://creativecommons.org/licenses/by/3.0/).

SUMMARY

Recent reports have shown that somatic cells, under appropriate culture conditions, could be directly reprogrammed to cardiac, hepatic, or neuronal phenotype by lineage-specific transcription factors. In this study, we demonstrate that both embryonic and adult somatic fibroblasts can be efficiently reprogrammed to clonal multilineage hematopoietic progenitors by the ectopic expression of the transcription factors ERG, GATA2, LMO2, RUNX1c, and SCL. These reprogrammed cells were stably expanded on stromal cells and possessed short-term reconstitution ability in vivo. Loss of p53 function facilitated reprogramming to blood, and p53$^{-/-}$ reprogrammed cells efficiently generated erythroid, megakaryocytic, myeloid, and lymphoid lineages. Genome-wide analyses revealed that generation of hematopoietic progenitors was preceded by the appearance of hemogenic endothelial cells expressing endothelial and hematopoietic genes. Altogether, our findings suggest that direct reprogramming could represent a valid alternative approach to the differentiation of embryonic stem cells (ESCs) or induced pluripotent stem cells (iPSCs) for disease modeling and autologous blood cell therapies.

INTRODUCTION

Until recently, it was assumed that differentiation was mostly a unidirectional and irreversible route that cells undertake during lineage commitment. This dogma was rebutted by the groundbreaking discovery of Yamanaka and colleagues that the expression of four transcription factors (TFs) could reprogram mouse and human cells into a pluripotent stage (Takahashi and Yamanaka, 2006; Takahashi et al., 2007). Subsequent studies have established that cellular-fate conversion is also obtained by direct transdifferentiation between two distinct lineages. Transdifferentiation is generally achieved by overexpressing lineage-instructive TFs, as demonstrated by the effective cell-fate switching of fibroblasts into neuronal, hepatocyte, and cardiomyocyte lineages (Du et al., 2014; Huang et al., 2014; Ieda et al., 2010; Nam et al., 2013; Sekiya and Suzuki, 2011; Vierbuchen et al., 2010).

The hematopoietic system relies on the existence of a rare population of hematopoietic stem cells (HSCs) that are able to self-renew and reconstitute the entire system by generating all hematopoietic lineages. In the clinic, transfusion of HSCs and terminally differentiated blood cells (erythrocytes, platelets, and granulocytes) is used to successfully treat blood genetic disorders and malignancies. However, a major restriction to the wider use of these treatments is the limited availability of cells from donors with adequate match. An alternative strategy for the generation of patient-specific hematopoietic cells would be to differentiate induced pluripotent stem cells (iPSCs) to HSCs. Unfortunately, so far, the development of robust methods to produce blood cells and, in particular, transplantable long-term HSCs has met with limited success (Blum and Benvenisty, 2008; Sturgeon et al., 2013). Therefore, direct reprogramming of patient-derived cells by transdifferentiation represents an attractive alternative strategy for the generation of transplantable blood cells.

Hematopoiesis is governed by the combined functions of numerous TFs, complicating attempts to establish simple approaches toward transdifferentiation into this lineage. This complexity is highlighted by knockout studies that identified multiple regulators of blood cell generation including SCL, RUNX1, ERG, and GATA2 (Loughran et al., 2008; Okuda et al., 1996; Robb et al., 1996; Tsai et al., 1994). Genome-wide chromatin immunoprecipitation data indicated that these four factors, in conjunction with LMO2, LYL1, and FLI1, create a regulatory complex that mediates transcription of multiple genes in hematopoietic progenitor cells (Wilson et al., 2010). Each TF of this heptad has been shown to act at multiple stages of hematopoietic specification, maturation, and differentiation (Loose et al., 2007). For example, SCL is required during the formation of hemogenic endothelium precursors from hemangioblast and mesoderm (Lancrin et al., 2010). RUNX1 is critical for the emergence of hematopoietic progenitors and HSCs from hemogenic endothelium (Chen et al., 2009; Lancrin et al., 2010). ERG is required for the maintenance of fetal HSCs and also for the

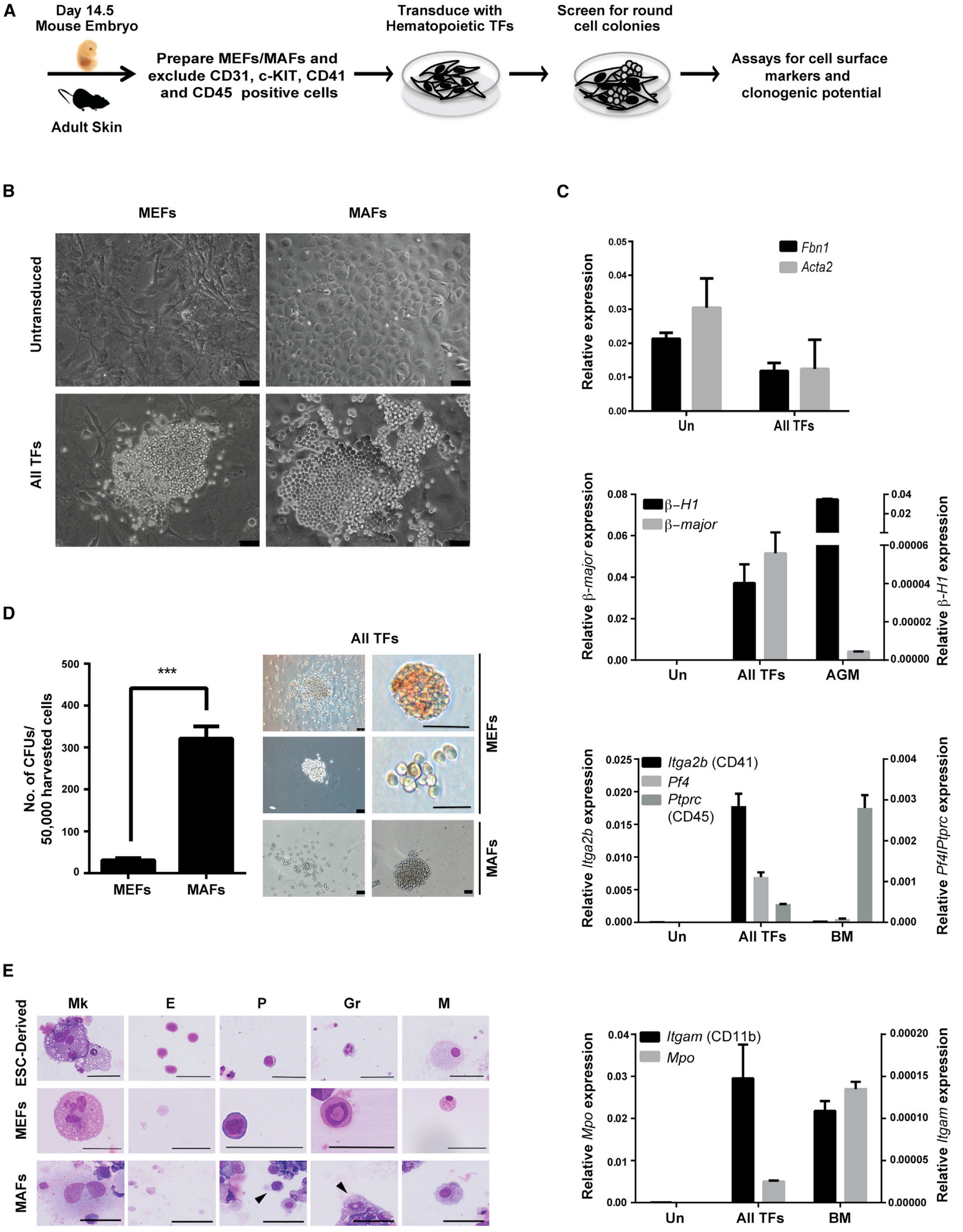

(legend on next page)

self-renewal and survival of adult HSCs (Loughran et al., 2008; Taoudi et al., 2011). Synergistic, antagonistic, and sequential relationships among these TFs create complex regulatory landscapes that shape the hematopoietic identity (Pimanda and Göttgens, 2010).

Previous studies have revealed an inherent plasticity of hematopoietic cells, as they are amenable to transdifferentiation and dedifferentiation. Recently, this approach has been remarkably employed for the generation of inducible HSCs (iHSCs) by reprogramming blood cells or endothelium (Riddell et al., 2014; Sandler et al., 2014). In both studies, cells capable of multilineage long-term engraftment were obtained by transient ectopic expression of TFs selectively expressed in HSPCs. Importantly, the successful generation of iHSCs required provision of a favorable niche for the maturation of the cells in the form of either the in vivo bone marrow environment or a vascular support mimicking the aorta-gonad-mesonephros (AGM) niche. However, reprogramming of differentiated blood cells might not be suitable for the generation of healthy transplantable cells for patients with blood malignancies or acquired genetic diseases (Pereira et al., 2014). In addition, it could prove very difficult to obtain enough endothelial cells from an adult patient to perform reprogramming. An approach more appropriate for this purpose, but also more challenging, would be to reprogram more developmentally distinct cell types, such as fibroblasts, into blood. In this context, it has been shown that the TFs PU.1 and cEBPα were capable of reprogramming fibroblasts into mature macrophage-like cells (Feng et al., 2008). However, reprogramming of fibroblasts to more immature hematopoietic progenitors has so far remained challenging. The first attempt, reported in 2010, involved the ectopic expression of the pluripotency factor OCT4 in human fibroblasts (Szabo et al., 2010). Although OCT4-induced hematopoietic progenitors exhibited myeloid and erythroid potential, lymphoid potential and long-term in vivo engraftment capacity were not achieved. Moreover, the use of the pluripotent TF OCT4 also raises concerns about tumorigenicity, as partial reprogramming induced by OCT4 could mimic a neoplastic state (Ohnishi et al., 2014). More recently, the combinatorial expression of a limited set of hematopoietic TFs, including GATA2, GFI1b, ETV6, and c-FOS in fibroblasts, was shown to induce a hemogenic endothelial cell fate (Pereira et al., 2013). However, the reprogrammed cells subsequently displayed only minimal hematopoietic potential despite coculture with placental cells.

In this report, we establish that fibroblasts can be rapidly and robustly reprogrammed to hematopoietic progenitors. The ectopic expression of five hematopoietic TFs, functionally selected from a range of 19 regulators, reproducibly induced hematopoietic fate in adult and embryonic fibroblasts within 8 days. The reprogrammed progenitors exhibited multilineage clonogenic capacity in vitro. Genome-wide transcriptional analyses of the reprogrammed cells revealed that the generation of hematopoietic progenitors was preceded by a hemogenic endothelial stage.

RESULTS

Reprogramming of Fibroblasts to Hematopoietic Fate with a Pool of Hematopoietic TFs

The aim of the present study was to investigate whether the ectopic expression of specific hematopoietic TFs could result in a direct and rapid cell fate conversion to hematopoietic precursors. First, we carefully selected a set of 19 different hematopoietic TFs based on their expression and function during hematopoiesis (Table S1). The expression of most of the selected TFs spans from the onset of blood development during ontogeny to the adult blood system (Orkin and Zon, 2008). Lentiviruses expressing each individual factor were prepared and a cocktail of all TFs was used to infect primary fibroblasts. As a source of starting material, we used either embryonic day 14.5 (E14.5) mouse embryonic fibroblasts (MEFs) or mouse adult ear skin fibroblasts (MAFs). Prior to infection, these cells were depleted of any CD41-, CD31-, c-KIT-, and CD45-positive cells to eliminate potential contamination by hematopoietic and endothelial cells (Figure 1A). Posttransduction, the infected cells were switched to a media supporting the growth of hematopoietic cells, and the appearance of colonies containing round cells was monitored daily. Starting from day 8, we observed in transduced MEFs and MAFs cultures, but not in untransduced control cells, the emergence of colonies of small round cells often associated with cobblestone-like areas (Figure 1B). These colonies continued to expand during the following weeks of culture.

The transduced cells were next tested by immunostaining for the acquisition of the hematopoietic cell-surface markers c-KIT and CD41, which are expressed on emerging blood cells during embryonic development (Mikkola et al., 2003; Mitjavila-Garcia et al., 2002), and for the expression of hematopoietic genes. Live staining at day 12 indicated that cells in emerging colonies

Figure 1. Screen for Hematopoiesis-Inducing TFs

(A) Schematic representation of experimental strategy. Murine embryonic fibroblasts (MEFs) were prepared from day 14.5 embryos, and murine adult fibroblasts (MAFs) were prepared from adult ear skin. Cells expressing the surface markers CD31, CD41, c-KIT, and CD45 were excluded from the starting populations. Sorted cells were transduced with a cocktail of all TFs. After 21 days of culture, cells were analyzed for hematopoietic cell-surface markers, clonogenic capacity, and cellular and nuclear morphology.

(B) Bright-field images of untransduced and all TFs transduced MEFs and MAFs at day 12.

(C) Relative gene expression levels of indicated genes with respect to β-actin in untransduced (Un), all-TF-transduced MEFs, control bone marrow (BM), and control E10.5 aorta-gonad-mesonephros (AGM) region cells. Data presented are representative of one out of three independent experiments performed in triplicate (n = 3; mean ± SD).

(D) Number of hematopoietic colonies generated by 50,000 all-TF-transduced day 21 harvested MEFs/MAFs (left). Mean ± SEM from two independent experiments performed in triplicates is shown (n = 2). Representative bright-field images of the different types of colonies observed (right).

(E) Cellular morphology of day 21 transduced MEFs/MAFs and ESC-derived hematopoietic cells. Arrowheads depict indicated morphologies.

Gr, granulocyte; M, macrophage; P, progenitor; E, erythrocyte; Mk, megakaryocyte, Scale bars represent 50 μm. Asterisks indicate significant differences (Student's t test; ***p < 0.0005).

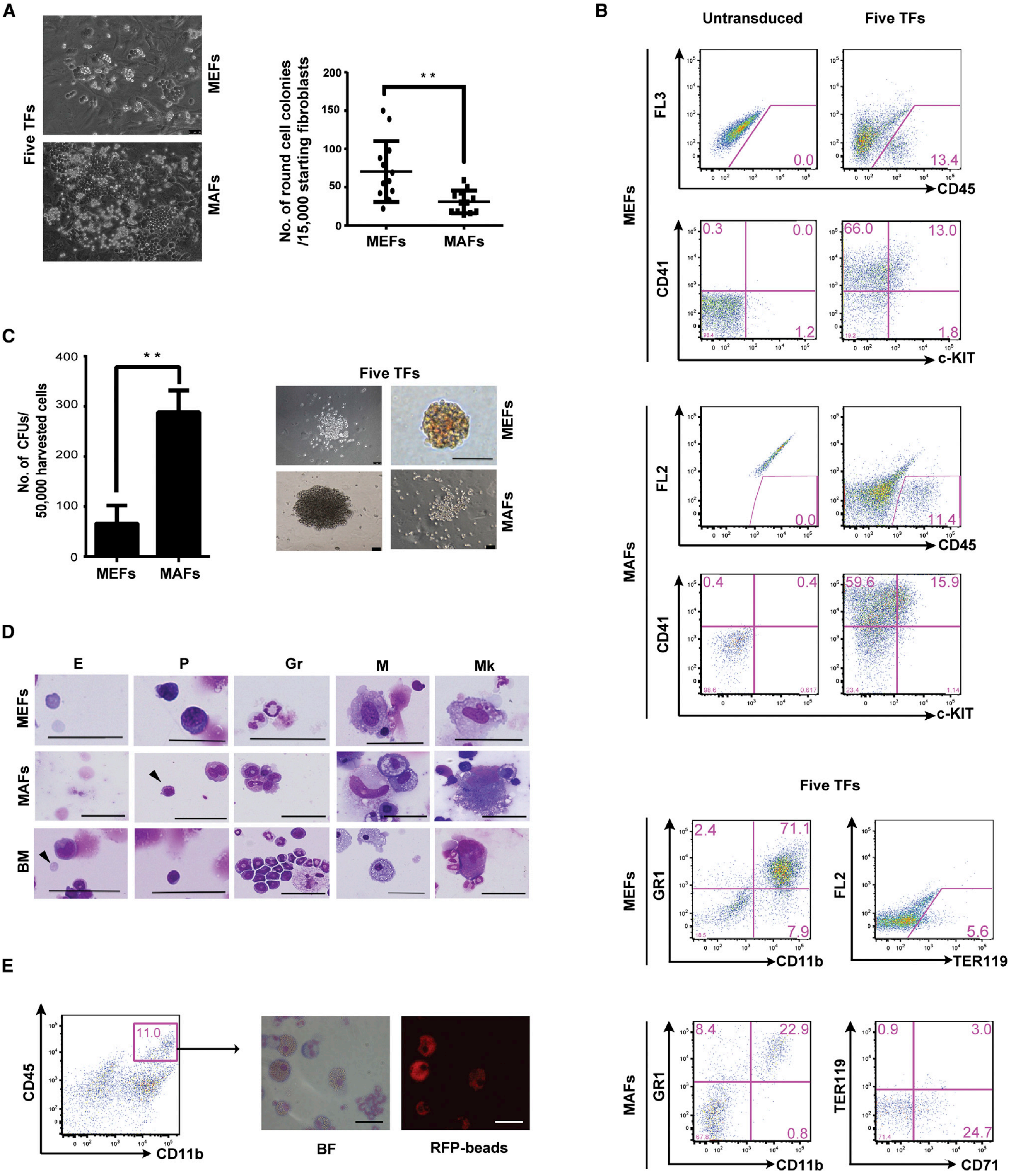

Figure 2. Five TFs Induce Reprogramming to Blood

(A) Bright-field images of five TFs transduced MEFs/MAFs at day 12 (left). Number of round cell colonies observed per 15,000 transduced MEFs/MAFs (right, n = 5, mean ± SEM).

(B) FACS analysis of untransduced and five-TF-transduced MEFs and MAFs at day 21.

(legend continued on next page)

were positive for CD41 expression (Figure S1A). Flow cytometry (fluorescence-activated cell sorting [FACS]) analyses at day 21 confirmed the acquisition of CD41 and low levels of c-KIT expression by the transduced MEFs and MAFs (Figure S1B). Gene expression analyses on 3-weeks-posttransduced cells clearly indicated the downregulation of the fibroblasts markers (*Acta2* and *Fbn1*) (Figure 1C). In contrast, the expression of genes associated with erythroid (embryonic *β-H1* and adult *β-major* hemoglobin), megakaryocytic (*Pf4*), and myeloid (*Itgam* and *Mpo*) lineages were markedly upregulated in these cultures (Figure 1C). The expression of both *β-H1* and *β-major* hemoglobin genes suggests the presence of both primitive and definitive erythroid cells in these cultures. Having demonstrated the induction of a hematopoietic gene signature, we next sought to determine whether the reprogrammed cells possessed functional hematopoietic clonogenic potential. Day 21 reprogrammed MEFs and MAFs, but not untransduced fibroblasts, generated hematopoietic colonies containing macrophage, erythroid, and granulocytic cells upon replating in clonogenic assays (Figure 1D). May-Grünwald Giemsa staining confirmed the presence of cells with erythroid, megakaryocytic, and myeloid morphologies in these colonies (Figure 1E). Collectively, these experiments established that both embryonic and adult fibroblasts could be reprogrammed to hematopoietic cells upon the ectopic expression of 19 hematopoietic TFs.

ERG, GATA2, LMO2, RUNX1c, and SCL Reprogram Fibroblasts to Blood

Our next aim was to define the minimal combination of TFs required for reprogramming to blood cells. By eliminating each TF individually from the pool, taking into account the redundancy among TFs, and reiteration, we established that a minimal set of five TFs (ERG, GATA2, LMO2, RUNX1c, and SCL) was able to robustly and reproducibly induce the generation of colonies of round cells in both adult and embryonic fibroblasts (Figure 2A, left; Movie S1). The efficiency of reprogramming was consistently higher in MEFs than in MAFs (Figure 2A, right). Several other combinations of TFs could also induce the generation of hematopoietic colonies from MEFs, albeit with lower efficiencies. The combination of ERG, FLI1, GATA2, PU.1, and SCL or the triad of FLI1, GATA2, and SCL, which controls the specification of mammalian hematopoietic progenitors (Pimanda et al., 2007), induced the generation of hematopoietic colonies in MEFs (Figure S2A). However, as the combination of the five TFs efficiently and reproducibly reprogrammed both MEFs and MAFs, we selected this set for further experiments. FACS analyses of day 21 MEF and MAF transduced cultures confirmed the acquisition of the hematopoietic markers c-KIT, CD41, CD45, CD11b, and TER119 (Figure 2B). Clonogenic assays and cytospin analysis of day 21 reprogrammed cells confirmed that five-TF-transduced MEFs and MAFs exhibited erythroid and myeloid potential (Figures 2C and 2D). Finally, sorted CD45/CD11b double-positive cells were able to uptake red fluorescent latex beads, demonstrating their phagocytic capacity and functionality (Figure 2E).

We next set out to determine the specific requirement for each TF and therefore performed "*N* minus 1" experiments. In MAFs, SCL, LMO2, and RUNX1c were more important than ERG and GATA2 for the generation of hematopoietic colonies (Figure S2B). In MEFs, SCL and LMO2 alone were sufficient, albeit at a lower efficiency, to generate hematopoietic colonies containing CD41-, CD45-, and c-KIT-positive cells (Figure S2C); in contrast, no colonies were obtained with these two TFs in MAFs (Figure S2C). To investigate if the hematopoietic phenotype of the reprogrammed cells was dependent on the sustained expression of the exogenous TFs, we performed quantitative RT-PCR (qRT-PCR) on day 4 and day 21 transduced MEFs with primers specific for exogenous transduced factors. These experiments demonstrated that the vector-driven transcription of all five TFs was silenced by day 21 (Figure S2D). In contrast, vector integration of all five viruses was enriched upon the emergence and expansion of blood cells in these cultures (Figure S2E). These results established the contribution of all five exogenous factors in the induction of the hematopoietic program. In addition, qRT-PCR specific for the endogenous genes indicated that expression of all five endogenous TFs was induced during reprogramming (Figure S2F). Finally, to determine the growth factors and cytokines essential to reprogram fibroblasts, we performed "*N* minus 1" experiments with MEFs. We observed that interleukin-3 (IL-3) was absolutely critical for reprogramming (Figure S2G), a finding consistent with its established role in the maintenance of hematopoietic progenitor cells. Collectively, our results indicate that the combination of ERG, GATA2, LMO2, RUNX1c, and SCL efficiently induces reprogramming of both embryonic and adult fibroblasts to blood.

Five-TF-Induced Reprogramming Generates Multipotent Progenitors

Both morphological analysis and cell-surface staining data suggested that a large fraction of day 21 reprogrammed hematopoietic cells were already mature and differentiated at this stage (Figure 2D). To investigate whether fibroblasts were reprogrammed through a transient hematopoietic progenitor stage, or transdifferentiated more directly to mature blood cells, we investigated the emergence of cells positive for hematopoietic precursor markers by FACS. We observed from day 10 onward a sudden rise in the frequency of c-KIT/CD41 double-positive cells followed by a similar rise in the emergence of $CD45^+$ cells from day 12 (Figure 3A). Time-course replating of transduced MEF cultures in semisolid colony assays indicated a transient peak of clonogenic potential around day 12–15 (Figure 3B), which correlated with higher frequencies of c-KIT^+ cells. A similar gradual increase in the number of c-KIT^+ cells and peak of clonogenic potential was observed in transduced MAF cultures (Figures S3A and S3B).

(C) Number of hematopoietic colonies generated by 50,000 five-TF-transduced day 21 harvested MEFs/MAFs (left, n = 4, mean ± SEM). Representative bright-field images of the different types of colonies observed (right).

(D) Cellular morphology analyses of day 21 transduced MEFs/MAFs and control bone marrow (BM) derived cells. Arrowheads depict indicated morphology. Gr, granulocyte; M, macrophage; P, progenitor; E, erythrocyte; Mk, megakaryocyte. Scale bars represent 50 μm.

(E) Phagocytic capacity of CD45/CD11b double-positive cells. Asterisks indicate significant differences (Student's t test; **p < 0.01).

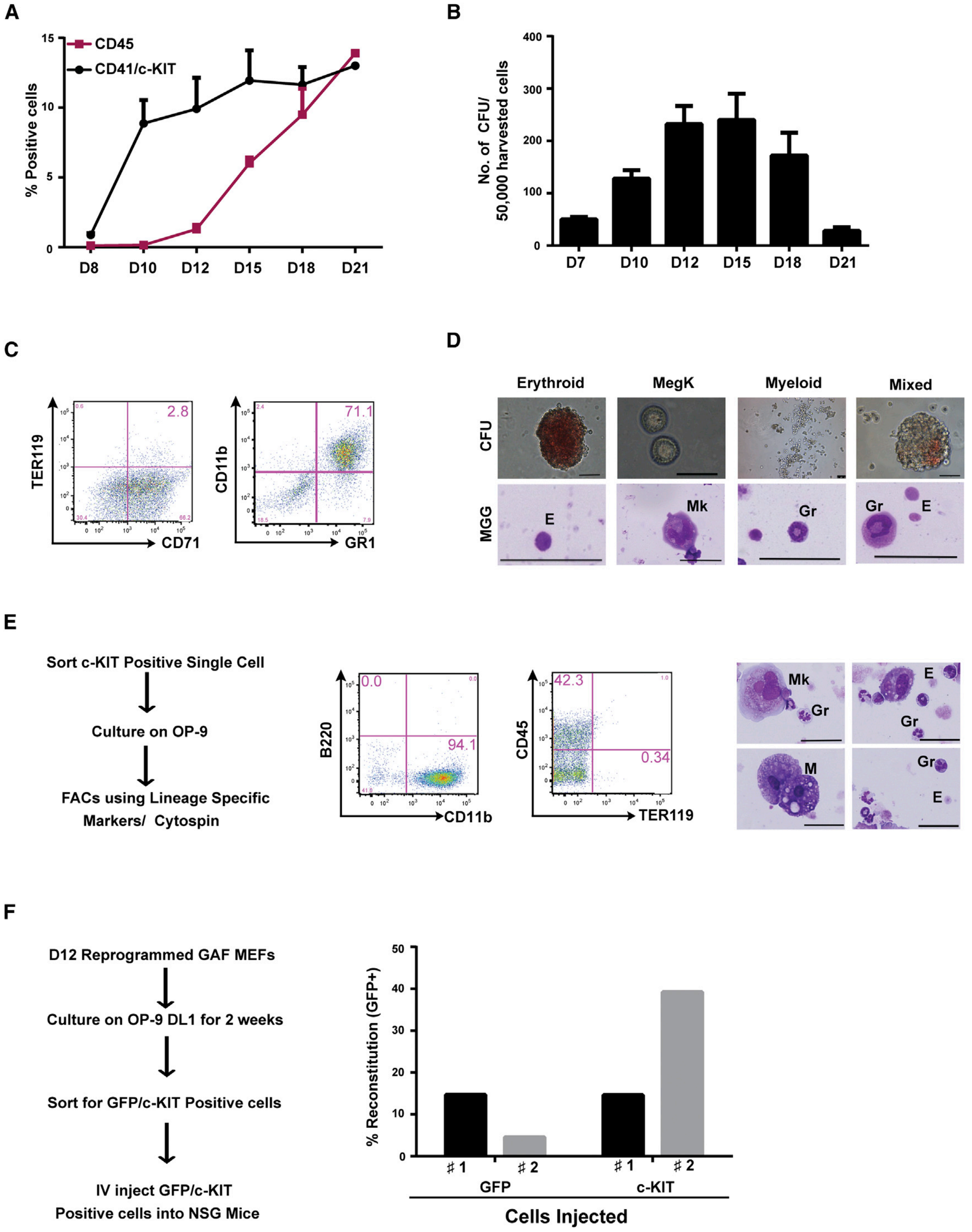

Figure 3. Multilineage Potential of Five-TF-Reprogrammed Cells

(A) Acquisition of hematopoietic cell surface markers during the course of reprogramming. Average percentages of cells expressing CD41, c-KIT, and CD45 are represented at indicated intervals after five-TF transduction of MEFs (n = 2, performed in duplicate; mean ± SD).

(B) Number of hematopoietic colonies generated by 50,000 reprogrammed MEFs from day 7 to day 21 (n = 2, performed in duplicate; mean ± SEM).

(C and D) Multilineage potential of five TFs reprogrammed day 12 sorted c-KIT$^+$ cells. (C) Sorted c-KIT$^+$ cells were cultured under erythroid and myeloid culture conditions for 1 week and FACS analyzed for cell-surface markers. (D) Morphology of colonies and cells generated by sorted c-KIT$^+$ cells.

(E) FACS and cellular morphology of cells derived from day 12 sorted single c-KIT$^+$ cell expanded on OP9 for 2 weeks (n = 3).

(F) Percentage reconstitution in peripheral blood determined by detection of donor-derived GFP$^+$ cells after 2 weeks of transplantation in two individual mice per group (1 and 2) either with GFP$^+$ or c-KIT$^+$ reprogrammed cells.

Confirming the relationship between c-KIT acquisition and clonogenic potential, c-KIT$^+$ cells displayed a higher clonogenic potential than c-KIT$^-$ cells (data not shown). When c-KIT$^+$ sorted cells were cultured in conditions that specifically support the growth of erythroid and myeloid cells, CD71/TER119 and CD11b/GR1 double-positive cells were respectively detected (Figure 3C). When evaluated in clonogenic replating assays, c-KIT$^+$ cells generated colonies containing erythroid, megakaryocytic, and myeloid cells (Figure 3D). Relatively similar genomic integration levels of the five vectors were observed between erythroid TER119 and myeloid CD11b-positive cells (Figure S3C). This observation suggests that both lineages have similar TF requirements for their generation and/or that they are generated through a common progenitor. Collectively, these results indicate that hematopoietic progenitors with myeloid, erythroid, and megakaryocytic potential are generated early during reprogramming.

To investigate the presence of multipotential progenitors, we evaluated the frequency of clonogenic precursors in the c-KIT$^+$ fraction, containing all hematopoietic progenitor cells, including the most immature precursors. Limiting dilution analyses indicated that 1 in 20 c-KIT$^+$ cells generated colonies when cultured on OP-9 stromal cells (data not shown). To investigate if c-KIT$^+$ cells have multilineage clonal ability, single day 12 fibroblast-derived c-KIT$^+$ cells were sorted onto OP-9 stromal cells and amplified for 2 weeks (Figure 3E). FACS and May-Grünwald Giemsa staining indicated the presence of erythroid, myeloid, and megakaryocytic cells in cultures initiated with a single c-KIT$^+$ cell (Figure 3E). These results suggest that fibroblasts are reprogrammed to the blood program, at least in part, through multipotential hematopoietic progenitor cells.

To further determine if hematopoietic stem cells might be generated during reprogramming, we performed FACS analyses for the SLAM markers on day 12 reprogrammed cells that were amplified on OP9-DL1. LIN$^-$c-KIT$^+$SCA1$^+$ (LSK) cells were detected in these cultures, and some of the LSK cells were CD48$^-$CD150$^+$, indicating the presence of phenotypic HSCs (Figure S3D). Additionally, we also observed phenotypic HSCs identified by the combination EPCR$^+$CD48$^-$CD45$^+$CD150$^+$ (Figure S4A). We next investigated if these reprogrammed cells have the capacity to engraft mice in vivo. For this, we established E14.5 MEFs from a transgenic mouse carrying the GFP reporter cDNA under the control of the pan-hematopoietic *AI467606* gene promoter (Ferreras et al., 2011). We amplified day 12 reprogrammed cells on OP9-DL1 stromal cells for 2 weeks. At this stage, GFP$^+$ cells, characterizing both progenitors and differentiated cells, or c-KIT$^+$ cells, including the most immature progenitors, were sorted and injected into two irradiated immunocompromised mice per each group. After 2 weeks, we monitored the presence of GFP$^+$ cells in the peripheral blood and detected short-term engraftment of both injected populations (Figures 3F and S4B). Engrafted cells were mostly TER119$^+$ erythroid cells (Figure S4B). However, 8 weeks postinjection, we could not detect engraftment greater than 1% in peripheral blood (data not shown). Collectively, these results suggest that the five-TF-mediated reprogrammed cells have short-term engraftment capacity and that further optimization of the culture conditions or TF composition will be necessary to obtain long-term engraftment capacity.

Loss of p53 Increases the Efficiency of Reprogramming to Blood

The loss of p53 or p16/p19 function has been shown to dramatically improve the efficiency of reprogramming to the pluripotent state (Hong et al., 2009; Li et al., 2009). We therefore investigated whether similarly deletion of these genes improved reprogramming of fibroblasts to blood. Following transduction with the five TFs, we observed significantly higher numbers of hematopoietic colonies generated by p53 and p16/p19-null MEFs than by wild-type MEFs (Figure 4A). These colonies emerged as early as 5 days after transduction, and scoring was performed on day 6, as their excessive proliferation precluded individual counting at later time points. Reprogramming of p53$^{-/-}$ MEFs resulted in the emergence of hematopoietic progenitors with multilineage potential (Figures 4B and 4C). Also, loss of p53 or p16/p19 function significantly increased the frequency of TER119$^+$ erythroid cells generated by reprogrammed cells (Figure 4D). Cells with mature megakaryocytic morphologies that stained positive for acetylcholinesterase activity were also more readily observed with p53$^{-/-}$ reprogrammed MEFs (Figure 4E). To evaluate the lymphoid potential of p53$^{-/-}$ reprogrammed cells, sorted c-KIT$^+$ cells were cultured and passaged on OP-9 or OP9-DL1 stromal cells in culture conditions supporting B or T cell growth, respectively. Cells positive for the B cell markers B220/CD19 emerged and proliferated during the culture of reprogrammed p53$^{-/-}$ MEFs (Figure 4Fi). A fraction of B220/CD19 double-positive reprogrammed p53$^{-/-}$ cells were also positive for immunoglobulin M expression (Figure 4Fi). The B cell identity of sorted B220/CD19-positive cells was further confirmed by detection of V(D)J chain rearrangements by PCR and sequencing (Figure 4Fii). Similarly, some reprogrammed p53$^{-/-}$ MEFs cells cultured on OP9-DL1 stromal cells acquired the expression of the early T cell marker CD25 and displayed T cell receptor rearrangements, confirming early T lymphoid commitment (Figures 4Gi and 4Gii). Collectively, these results demonstrate that p53$^{-/-}$ MEFs are more efficiently reprogrammed to hematopoietic progenitors with erythroid, myeloid, megakaryocyte, and B and T lymphoid lineage potential than wild-type MEFs, indicating that P53 expression is a barrier for reprogramming to blood.

Reprogramming to Blood Occurs via an Intermediate Endothelial Stage

We next investigated the molecular events and cellular processes leading to the reprogramming of fibroblasts to blood progenitors. The rapid emergence of hematopoietic colonies suggested that the reprogramming did not involve a pluripotent stem cell stage with subsequent differentiation into blood cells. Accordingly, the expression of pluripotent markers (*Oct4*, *Sox2*, and *Nanog*) was not detected during the course of reprogramming (Figure 5A). The detection of embryonic hemoglobin *β-H1* expression in reprogrammed cells (Figure 1C) suggested that the reprogramming process might recapitulate to some extent embryonic hematopoiesis. During embryonic hematopoiesis, mesodermal hemangioblast precursors generate primitive as well as definitive blood cells through an intermediate hemogenic endothelium stage. We evaluated by PCR whether the reprogramming of fibroblasts to blood process was associated

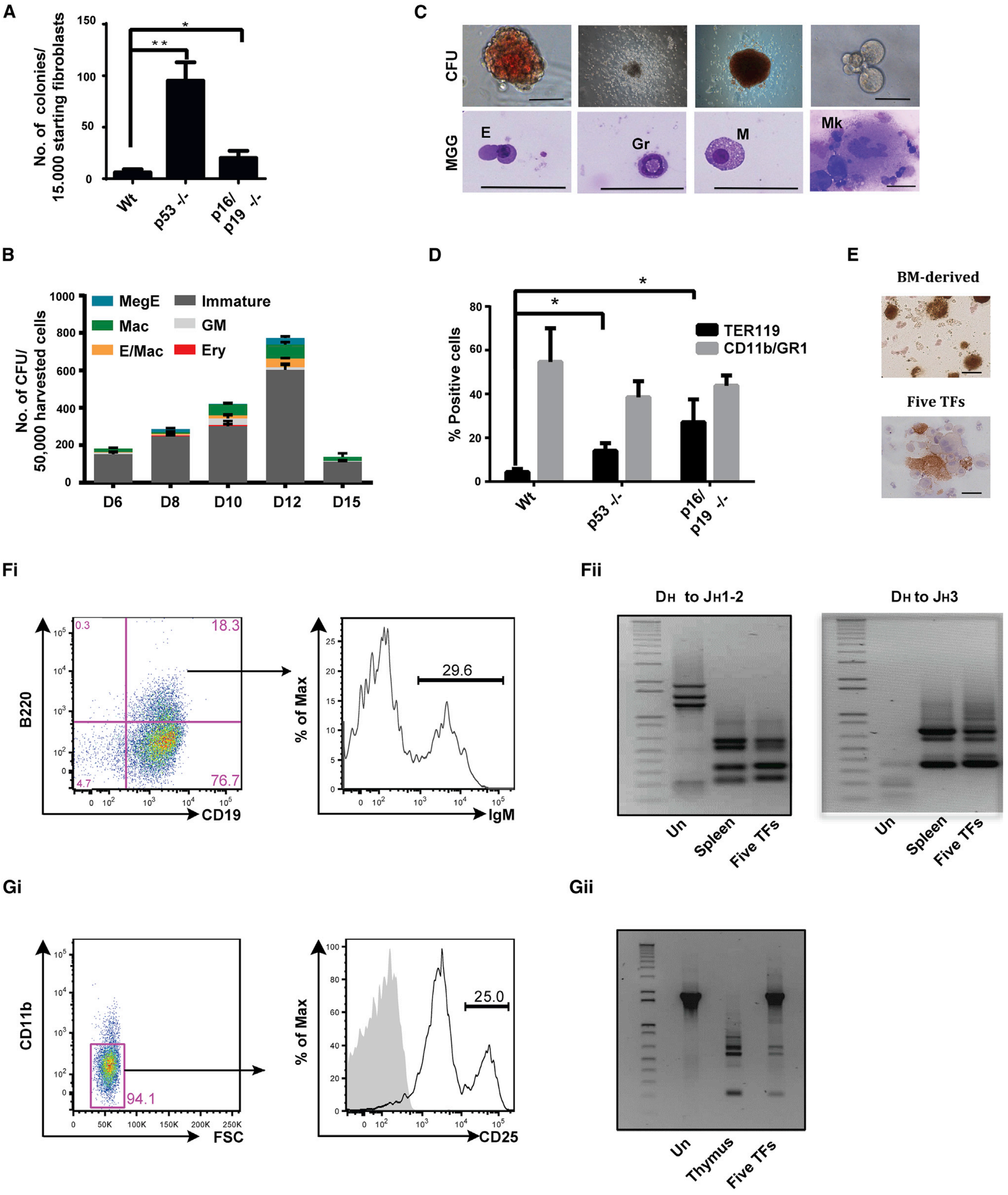

Figure 4. Reprogramming p53-Null MEFs

(A) Number of round cell colonies observed at day 6 per 15,000 transduced WT, $p53^{-/-}$, and $p16/p19^{-/-}$ MEFs (n = 4; mean ± SEM).

(B) Number and types of hematopoietic colonies generated by 50,000 five-TF-reprogrammed $p53^{-/-}$ MEFs harvested from day 6 to 15. Data presented are mean ± SEM of triplicates in a representative experiment (n = 2).

(legend continued on next page)

with any of these distinct embryonic steps. Although we did not detect any expression of the *Brachyury* mesodermal marker (data not shown), we clearly observed a peak of endothelial gene expression (*Cdh5*, *Tie2*, and *Icam1*; *Pecam1* and *Vwf*) early in reprogramming (Figures 5B and 5C). In contrast, hematopoietic genes (*Itga2b*, *Mpo*, and *Pu.1*) showed a delayed but steady increase in expression from day 12 to day 21 (Figure 5D). Live staining for vascular endothelial cadherin (CDH5) performed on day 5 cultures confirmed the early emergence of clusters of CDH5$^+$ endothelial cells (Figure 5E). To determine if these endothelial cells corresponded to hemogenic endothelium that gives rise to differentiated blood cells, we sorted CDH5$^+$ and CDH5$^-$ cells from c-KIT-depleted day 6 transduced MEFs to eliminate any potential hematopoietic precursors. The cells were then cultured in conditions that support the transition to hematopoietic progenitors before seeding them in clonogenic assays. As shown in Figure 5F, CDH5$^+$, but not CDH5$^-$, cells generated hematopoietic colonies, demonstrating their hemogenic potential. In addition, these c-KIT$^-$/CDH5$^+$ endothelial cells acquired the hematopoietic markers CD41, CD45, and c-KIT upon culture on OP9 (Figure S5A). We concluded from these results that reprogramming of fibroblasts to blood cells proceeds through an intermediate hemogenic endothelial cellular stage.

Transcriptome Analyses on Reprogrammed Cells

To define the global changes in gene expression driving reprogramming of fibroblasts to hematopoietic lineages, we performed exon array analyses on biological replicates of untreated MEFs (Un), day 8 sorted CDH5$^+$ cells, and day 12 sorted c-KIT$^+$ cells. We identified 868 significantly (5% false discovery rate [FDR]) differentially expressed genes (DEGs). Principal component analyses based on these DEGs confirmed the correlation between replicates and the differences between cells at the three stages of reprogramming (Figure S6A). We then performed hierarchical clustering on DEGs and observed three main clusters (Figure 6A). The first cluster mostly contained genes that were gradually downregulated from untransduced MEFs to CDH5$^+$ and then to c-KIT$^+$ populations and were associated with adhesion and muscular development (Figure 6A; Table S2). This cluster included fibroblast-specific genes (*Acta2*, *Actg2*, *Col2A*, *Col4a1*, *Col5a1*, and *Fgf3*) that were silenced during reprogramming. The second cluster encompassed genes that were upregulated in CDH5$^+$ cells but then downregulated in c-KIT$^+$ cells. These genes were mainly of endothelial nature and associated with blood vessel development and cell-cell adhesion (Figure 6A; Table S2). The third cluster included genes that were moderately expressed in CDH5$^+$ cells, highly expressed in c-KIT$^+$ populations, and associated with ontology terms such as development of the hematopoietic program, leukocyte migration, chemotaxis, and response to infection/wounding ontology terms (Figure 6A; Table S2). Pathway analyses with DAVID confirmed that hematopoietic pathways were already activated in the CDH5$^+$ fraction, a finding consistent with a hemogenic endothelium identity (Table S2). To further confirm this transient upregulation of the endothelial program, we performed gene set enrichment analyses (GSEA) with endothelial-specific genes. As expected, genes upregulated between MEFs and CDH5$^+$ cells, and downregulated between CDH5$^+$ and c-KIT$^+$ cells, were positively correlated with an endothelial gene signature (Figure 6B). These analyses also indicated an enrichment for genes specifically upregulated in CDH5$^+$ cells for early hematopoietic progenitors signature, whereas c-KIT$^+$-specific genes were more closely associated with late or differentiated hematopoietic progenitor cells (Figures 6C and S6B). Finally, we investigated to which extent our reprogrammed cells were similar to HSCs by comparing our transcriptome signature with previously published data sets from different HSC populations isolated from diverse hematopoietic tissues (McKinney-Freeman et al., 2012). We observed that CDH5$^+$ cells clustered with AGM, placental, and yolk sac HSCs that have been classified as specifying HSCs. Interestingly, c-KIT$^+$ cells clustered more closely with definitive HSCs from fetal liver, bone marrow, and embryonic stem cell (ESC)-derived HSCs (Figure 6D). Collectively, our transcriptome analyses further support the concept that reprogramming to hematopoietic progenitors proceeds through a hemogenic endothelium intermediate. This CDH5$^+$ cell population, while displaying clear evidence of its endothelial nature, also expressed hematopoietic genes and displayed similarity with emerging HSCs and early progenitors, whereas the later c-KIT$^+$ population was more closely associated with hematopoietic progenitors.

DISCUSSION

In this study, we establish that fibroblasts can be robustly reprogrammed to hematopoietic cell fate by concomitant ectopic expression of the hematopoietic TFs ERG, GATA2, LMO2, RUNX1c, and SCL. These five TFs have been shown to interact and act at diverse stages of the hematopoietic program; i.e., from the onset of its development to the more established adult hematopoietic hierarchy (Wilson et al., 2010). In particular, SCL, GATA2, and FLI1, a distinct but related-to-ERG ETS factor, form an interconnected regulatory triad that is activated during specification of HSCs (Pimanda et al., 2007). Once established, this circuit is self-maintained, providing the newly specified progenitors with a memory of their stemness in a similar fashion as OCT4, SOX2, and NANOG in pluripotent

(C) Morphology of colonies and cells obtained from five-TF-transduced day 12 sorted c-KIT$^+$ p53$^{-/-}$ MEFs.
(D) Average percentage of TER119 and CD11b/GR1 double-positive cells generated by day 12 sorted c-KIT$^+$ cells from WT, p53$^{-/-}$, and p16/p19$^{-/-}$ transduced MEFs (n = 3; mean ± SEM).
(E) Acetylcholinesterase staining of megakaryocytes derived from bone marrow or generated by day 12 sorted c-KIT$^+$ reprogrammed p53$^{-/-}$ MEFs.
(Fi) FACS analysis of c-KIT$^+$ sorted reprogrammed p53$^{-/-}$ MEFs after expansion on OP9 in lymphoid medium.
(Fii) BCR rearrangements detection in B220$^+$/CD19$^+$ sorted reprogrammed p53$^{-/-}$ MEFs, control untransduced MEFs (Un) and spleen cells.
(Gi) FACS analysis of c-KIT$^+$ sorted reprogrammed p53$^{-/-}$ MEFs after expansion on OP9-DL1 in lymphoid medium.
(Gii) TCR rearrangement detection in sorted CD25$^+$ reprogrammed p53$^{-/-}$ MEFs, control untransduced MEFs (Un) and thymus cells.
Asterisk(s) represents statistical significance (Student's t test; *p < 0.05, **p < 0.01).

OPEN ACCESS
CellPress

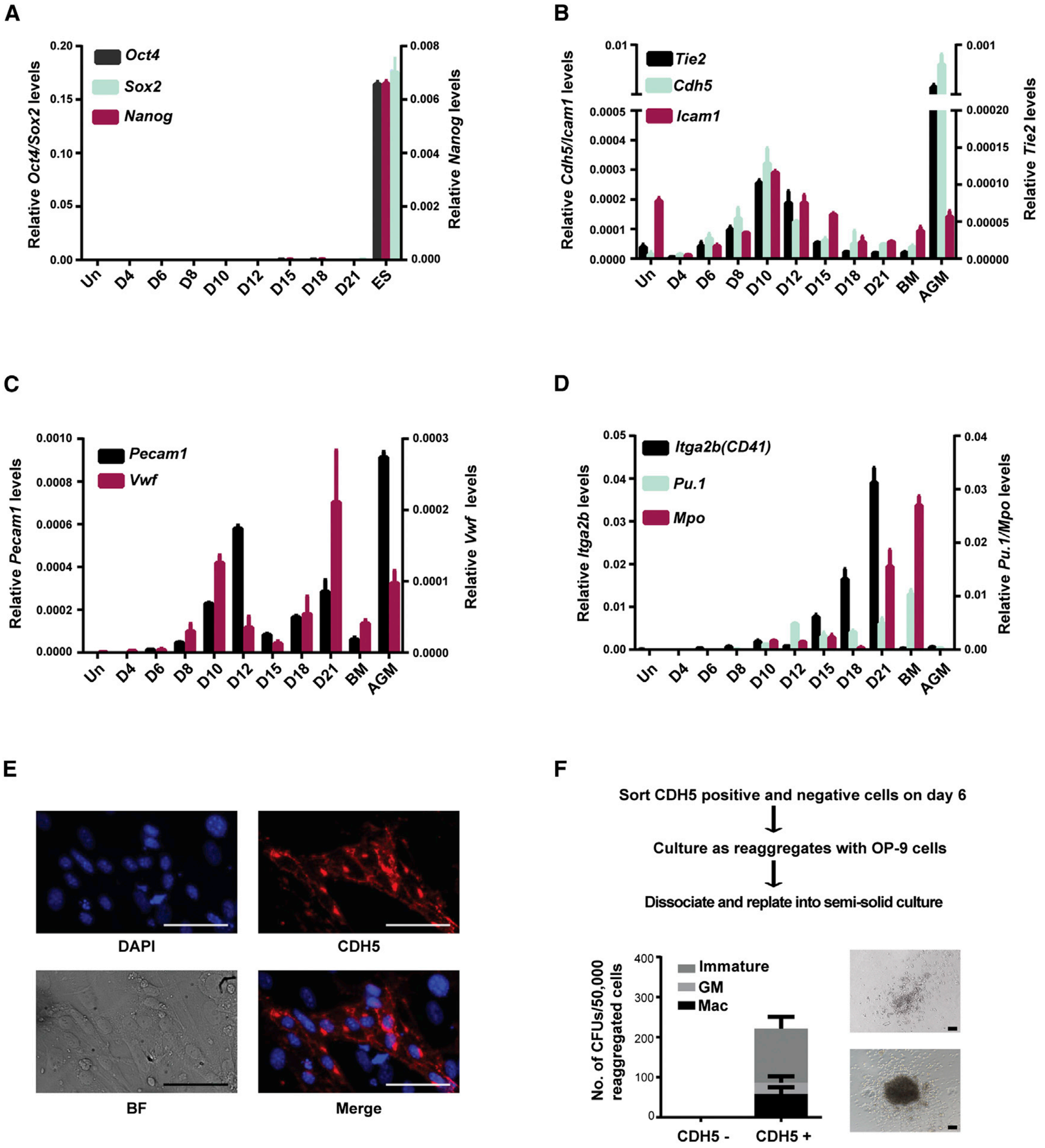

Figure 5. Fibroblasts Are Reprogrammed to Blood via an Intermediate Hemogenic Endothelial Stage

(A–D) Relative gene expression levels of pluripotent (A), endothelial (B), endothelial/ hematopoietic (C), and hematopoietic (D) markers with respect to β-actin at indicated days after five-TF transduction and in control bone marrow (BM) and aorta-gonad-mesonephros (AGM) region cells. Data presented are mean ± SD from one representative experiment (n = 3).

(E) Immunostaining of day 5 five-TF-transduced MEFs for CDH5 (red) and DAPI (blue).

(F) Schematic strategy to determine hemogenic potential of CDH5^{-}cKIT^{-} and CDH5^{+}cKIT^{-} five-TF-transduced cells (top). Number, type, and morphology of hematopoietic colonies generated by sorted cells reaggregated with irradiated OP9 stromal cells.

Data presented are mean ± SEM of triplicates of a representative experiment (n = 2). Scale bars represent 50 μm.

ESCs (Boyer et al., 2005). Furthermore, ERG, when expressed along with HOXA9 and RORA, has also been shown to confer multilineage potential to myeloid-restricted precursors (Doulatov et al., 2013). The transcription factor SCL and its binding partners are implicated in the induction of the hematopoietic program in mesoderm and the generation of hemogenic endothelium (Gering et al., 2003; Lancrin et al., 2009). Finally, RUNX1 physically interacts with SCL, ERG, and GATA2 and is critical for the transition from hemogenic endothelium to hematopoietic stem and progenitor cells (HSPCs) (Chen et al., 2009; Lancrin et al., 2010; Wilson et al., 2010). Furthermore, enforced expression of the isoform RUNX1a enhances

OPEN ACCESS
CellPress

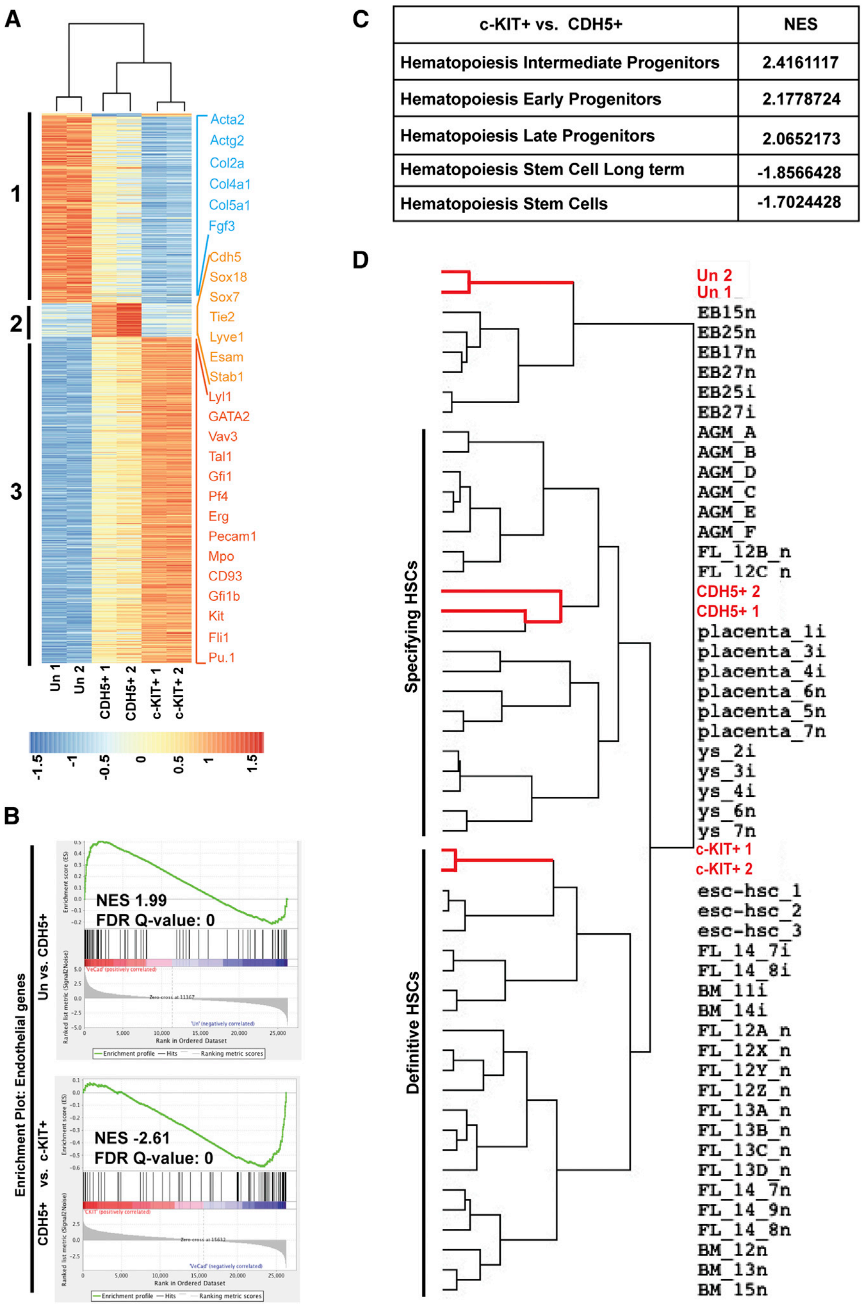

c-KIT+ vs. CDH5+	NES
Hematopoiesis Intermediate Progenitors	2.4161117
Hematopoiesis Early Progenitors	2.1778724
Hematopoiesis Late Progenitors	2.0652173
Hematopoiesis Stem Cell Long term	-1.8566428
Hematopoiesis Stem Cells	-1.7024428

Figure 6. Global Transcriptome Analyses of Reprogrammed Cells

Day 8 CDH5^{+} (CDH5-positive) and day 12 c-KIT^{+} (c-KIT-positive) flow-sorted cells were compared to untreated (Un) MEFs in duplicate by mouse Affymetrix exon arrays.

(A) Hierarchical clustering of differentially expressed genes (DEGs) among untreated MEFs, day 8 sorted CDH5^{+}, and day 12 sorted c-KIT^{+} cells. Genes specific to specified cell types are shown on the right.

(B) Gene set enrichment analysis (GSEA) for endothelial gene signature in untreated, CDH5^{+}, and c-KIT^{+} transcriptomes.

(C) Normalized enrichment score (NES) values obtained after performing GSEA for indicated gene sets comparing CDH5^{+} and c-KIT^{+} transcriptome data sets.

(D) Unsupervised hierarchical clustering of DEGs in our transcriptome data sets along with expression in HSCs from different hematopoietic organs (McKinney-Freeman et al., 2012).

hematopoietic lineage commitment from human ESCs and iPSCs (Ran et al., 2013). The close functional association among these five TFs during normal HSPCs generation could explain why we functionally identified them as the best combination of TFs to robustly induce a complex hematopoietic program in differentiated fibroblasts. Interestingly we also demonstrated that SCL and LMO2 are sufficient to induce hematopoietic fate in embryonic but not adult fibroblasts. This finding raises the interesting prospect that a limited set of small-molecule modulators could reinforce reprogramming or even circumvent the need for viral transduction.

We observed that the generation of blood precursors by the ectopic expression of our set of five TFs is a very fast process. The first morphological signs of cell-fate switching were observed by day 5 followed by the emergence and proliferation of c-KIT^{+} progenitors by 8 days. These cells exhibited a wide range of differentiation potential with robust generation of granulocytes and functional macrophages and, to a lower extent, erythrocytes and megakaryocytes. Furthermore, using fibroblasts with a p53$^{-/-}$ background broadened the range and increased the frequency of the generated lineages. In addition to larger pool of erythrocytes and megakaryocytes, we were able to generate B and T cells at different developmental stages. The results obtained with p53$^{-/-}$ MEFs provide a proof of principle that our combination of five TFs is suitable for reprogramming to all major blood lineages. The addition of small-molecule modulators that transiently increase epigenetic plasticity or briefly inhibit p53 or p16 might further extend the range and frequency of lineages generated from wild-type fibroblasts. In contrast, a permanent inactivation of p53 might not be desirable given the requirement for p53 to limit aberrant self-renewal (Zhao et al., 2010).

Establishing methods for transdifferentiation of nonhematopoietic cells to blood lineage remains challenging due to significant epigenetic barriers to overcome and the need to induce and maintain complex regulatory networks in these cells. So far, there are only two reports describing transdifferentiation of fibroblasts to blood progenitors. In the first study, hematopoietic progenitors with multilineage potential were generated

by overexpression of a single pluripotency factor, OCT4, in a hematopoietic-supportive culture environment (Szabo et al., 2010). The use of a pluripotent TF, however, raises some concerns about tumorigenicity. In the second report, Pereira et al. demonstrated that ectopic expression of c-FOS, ETV6, GATA2, and GFI1b reprogrammed fibroblasts to hemogenic endothelial cells, which upon further coculture with placental cells generated myeloid cells, albeit with a low efficiency (around 10 colonies for 100,000 starting MEFs) (Pereira et al., 2013). Although reprogramming by c-FOS, ETV6, GATA2, and GFI1b, as well as our combination of five TFs, seems to transit through a similar hemogenic endothelium intermediate stage, the efficiency and kinetics are quite different. While these authors identified a hemogenic endothelium population emerging by day 35 of reprogramming, our results indicate the emergence of an endothelial population as early as day 5. We detected c-KIT^{+} functional hematopoietic progenitors with multilineage potential as early as 8 days after transduction, whereas Pereira et al. obtained only limited myeloid potential after more than 6 weeks of culture. Collectively, these results indicate that our combination of TFs induce the rapid generation of a multipotent blood progenitors with better efficiency than previously reported.

Several lines of evidence suggest that the reprogramming of fibroblasts to blood cells recapitulates the embryonic process of blood development. CD41, the first hematopoietic marker we detected at the surface of transitioning reprogrammed cells, is widely expressed on the earliest blood progenitors generated during ontogeny (Figure S1A) (Ferkowicz et al., 2003; Mikkola et al., 2003; Mitjavila-Garcia et al., 2002; Robin et al., 2011). The detection of primitive erythrocytes expressing the embryonic βH1-hemoglobin also suggests that these TFs are inducing early developmental steps. Moreover, the presence of a transient hemogenic endothelium population resembles the endothelial-to-hematopoietic transition that takes place during blood development in the yolk sac or dorsal aorta (Boisset et al., 2010; Jaffredo et al., 1998; Lancrin et al., 2009; Zovein et al., 2008). Finally, the detection of phenotypic HSCs, as well as the gene expression profile clustering of the CDH5^{+} cell population with specifying HSC cell populations, further supports the hypothesis that the reprogramming mimics early hematopoietic development (Figures 6D, S3D, and S4A). The fact that we are recapitulating the normal embryonic program of blood development suggests that it may be only due to the limitations of the in vitro cultures that we cannot functionally identify an emerging HSC population. The reports by the Rossi and Rafii's laboratories emphasize the requirement for a supportive niche to obtain engraftable populations upon reprogramming of blood or endothelial cells (Riddell et al., 2014; Sandler et al., 2014). Providing a similar in vivo bone marrow or in vitro vascular support might greatly improve the repopulation potential of cells generated from fibroblasts and therefore will be a major direction for further research.

In conclusion, we provide a report of efficient and rapid reprogramming of fibroblasts to blood progenitors with a limited set of hematopoietic TFs. Fibroblasts represent an accessible and safe starting cell population for reprogramming. Despite many remaining questions, the approach reported here holds a huge promise and should be further optimized and developed for its safe use in clinical settings.

EXPERIMENTAL PROCEDURES

MEF and MAF Preparation

E14.5 MEFs were isolated as described previously (Sroczynska et al., 2009). MAFs were isolated from the ears of 2- to 6-month-old mice by collagenase digestion. Passage 0 fibroblasts were cultured until confluent and frozen. Before infection, MEFs and MAFs were depleted of cells positive for the hematopoietic/endothelial markers CD41, CD31, c-KIT, and CD45.

Lentivirus Transduction, Colony-Forming Unit Assay, and Reaggregation

MEFs/MAFs (15,000) were seeded on a gelatin-coated 12-well plate, and after 24 hr viral transductions were carried out in the presence of 10 μg/ml diethylaminoethyl dextran. After 4 hr of transduction, the cultures were placed in hematopoietic media (1× Iscove's modified Dulbecco's medium supplemented with plasma-derived serum [PDS; Antech], 10% protein-free hybridoma medium [PFM; GIBCO], 0.5 mM ascorbic acid, 4.5×10^{-4} M MTG, 2 mM L-glutamine, 80 mg/ml transferrin, 1% c-KIT ligand, 1% IL-3, 1% granulocyte-macrophage colony-stimulating factor, 1% thrombopoietin conditioned media, 4 U/ml erythropoietin [Ortho-Biotech], 10 ng/ml macrophage colony-stimulating factor, 10 ng/ml IL-6, 5 ng/ml IL-11 [all from R&D Systems], and 50 μg/ml penicillin-streptomycin). Colony-forming unit (cfu) assays were performed as described previously (Sroczynska et al., 2009). To evaluate the presence of hemogenic endothelium, CDH5^{+} and CDH5^{-} cells were sorted from day 6 cultures that were depleted for c-KIT^{+} cells to avoid hematopoietic contamination. A total of 20,000 sorted cells were mixed with 100,000 irradiated OP9 cells, cultured overnight in hanging-drop cultures, and then on Durapore filter (Millipore) for another 4 days. Single-cell suspensions obtained following dissociation with collagenase/dispase solution were assayed in cfu assays.

Transplantation Assay

E14.5 MEFs for transplantation experiments were obtained from a reporter mouse line with GFP expression under the control of *AI467606* promoter (Ferreras et al., 2011). Day 12 transduced cells were harvested and further expanded for 2 weeks on OP-DL1 stroma cells in hematopoietic medium. GFP- or c-KIT-positive cells were sorted and intravenously injected in 6- to 8-week-old lethally irradiated NSG mice (n = 2). The level of engraftment was determined by GFP expression in peripheral blood. All animal work was performed under regulations set out by the Home Office Legislation under the 1986 United Kingdom Animal Scientific Procedures Act.

Affymetrix Analyses and Integration of Data Sets

Global gene expression analyses were done using the Mouse Exon 1.0 ST arrays. The R/Bioconductor package LIMMA was used to identify genes that were differentially expressed among the three conditions. Differentially expressed genes were obtained with significant p value and at 5% FDR. The distance weighted discrimination (Benito et al., 2004) method for cross-platform normalization was done for our transcriptome data sets along with McKinney data sets (McKinney-Freeman et al., 2012). Hierarchical clustering was performed using the complete linkage.

ACCESSION NUMBERS

Affymetrix data were deposited in the Gene Expression Omnibus under the accession number GSE59428.

SUPPLEMENTAL INFORMATION

Supplemental Information includes Supplemental Experimental Procedures, six figures, two tables, and one movie and can be found with this article online at http://dx.doi.org/10.1016/j.celrep.2014.11.002.

AUTHOR CONTRIBUTIONS

K.B. and M.F. designed and performed experiments, analyzed the data, and wrote the manuscript. V.K. and G.L. designed and supervised the research project, analyzed the data, and wrote the manuscript.

ACKNOWLEDGMENTS

We thank the staff at the Advanced Imaging, Animal Facility, Molecular Biology Core facilities, and Flow Cytometry of CRUK Manchester Institute for technical support and Hui Sun Leong for help with the bioinformatic analyses. We thank members of the Stem Cell Biology and Stem Cell Haematopoiesis group for valuable advice and critical reading of the manuscript. Work in our laboratory is supported by the Leukaemia and Lymphoma Research Foundation, the Biotechnology and Biological Sciences Research Council, and Cancer Research UK.

Received: July 1, 2014
Revised: October 10, 2014
Accepted: November 4, 2014
Published: November 26, 2014

REFERENCES

Benito, M., Parker, J., Du, Q., Wu, J., Xiang, D., Perou, C.M., and Marron, J.S. (2004). Adjustment of systematic microarray data biases. Bioinformatics *20*, 105–114.

Blum, B., and Benvenisty, N. (2008). The tumorigenicity of human embryonic stem cells. Adv. Cancer Res. *100*, 133–158.

Boisset, J.-C., van Cappellen, W., Andrieu-Soler, C., Galjart, N., Dzierzak, E., and Robin, C. (2010). In vivo imaging of haematopoietic cells emerging from the mouse aortic endothelium. Nature *464*, 116–120.

Boyer, L.A., Lee, T.I., Cole, M.F., Johnstone, S.E., Levine, S.S., Zucker, J.P., Guenther, M.G., Kumar, R.M., Murray, H.L., Jenner, R.G., et al. (2005). Core transcriptional regulatory circuitry in human embryonic stem cells. Cell *122*, 947–956.

Chen, M.J., Yokomizo, T., Zeigler, B.M., Dzierzak, E., and Speck, N.A. (2009). Runx1 is required for the endothelial to haematopoietic cell transition but not thereafter. Nature *457*, 887–891.

Doulatov, S., Vo, L.T., Chou, S.S., Kim, P.G., Arora, N., Li, H., Hadland, B.K., Bernstein, I.D., Collins, J.J., Zon, L.I., and Daley, G.Q. (2013). Induction of multipotential hematopoietic progenitors from human pluripotent stem cells via respecification of lineage-restricted precursors. Cell Stem Cell *13*, 459–470.

Du, Y., Wang, J., Jia, J., Song, N., Xiang, C., Xu, J., Hou, Z., Su, X., Liu, B., Jiang, T., et al. (2014). Human hepatocytes with drug metabolic function induced from fibroblasts by lineage reprogramming. Cell Stem Cell *14*, 394–403.

Feng, R., Desbordes, S.C., Xie, H., Tillo, E.S., Pixley, F., Stanley, E.R., and Graf, T. (2008). PU.1 and C/EBPalpha/beta convert fibroblasts into macrophage-like cells. Proc. Natl. Acad. Sci. USA *105*, 6057–6062.

Ferkowicz, M.J., Starr, M., Xie, X., Li, W., Johnson, S.A., Shelley, W.C., Morrison, P.R., and Yoder, M.C. (2003). CD41 expression defines the onset of primitive and definitive hematopoiesis in the murine embryo. Development *130*, 4393–4403.

Ferreras, C., Lancrin, C., Lie-A-Ling, M., Kouskoff, V., and Lacaud, G. (2011). Identification and characterization of a novel transcriptional target of RUNX1/AML1 at the onset of hematopoietic development. Blood *118*, 594–597.

Gering, M., Yamada, Y., Rabbitts, T.H., and Patient, R.K. (2003). Lmo2 and Scl/Tal1 convert non-axial mesoderm into haemangioblasts which differentiate into endothelial cells in the absence of Gata1. Development *130*, 6187–6199.

Hong, H., Takahashi, K., Ichisaka, T., Aoi, T., Kanagawa, O., Nakagawa, M., Okita, K., and Yamanaka, S. (2009). Suppression of induced pluripotent stem cell generation by the p53-p21 pathway. Nature *460*, 1132–1135.

Huang, P., Zhang, L., Gao, Y., He, Z., Yao, D., Wu, Z., Cen, J., Chen, X., Liu, C., Hu, Y., et al. (2014). Direct reprogramming of human fibroblasts to functional and expandable hepatocytes. Cell Stem Cell *14*, 370–384.

Ieda, M., Fu, J.D., Delgado-Olguin, P., Vedantham, V., Hayashi, Y., Bruneau, B.G., and Srivastava, D. (2010). Direct reprogramming of fibroblasts into functional cardiomyocytes by defined factors. Cell *142*, 375–386.

Jaffredo, T., Gautier, R., Eichmann, A., and Dieterlen-Lièvre, F. (1998). Intraaortic hemopoietic cells are derived from endothelial cells during ontogeny. Development *125*, 4575–4583.

Lancrin, C., Sroczynska, P., Stephenson, C., Allen, T., Kouskoff, V., and Lacaud, G. (2009). The haemangioblast generates haematopoietic cells through a haemogenic endothelium stage. Nature *457*, 892–895.

Lancrin, C., Sroczynska, P., Serrano, A.G., Gandillet, A., Ferreras, C., Kouskoff, V., and Lacaud, G. (2010). Blood cell generation from the hemangioblast. J. Mol. Med. *88*, 167–172.

Li, H., Collado, M., Villasante, A., Strati, K., Ortega, S., Cañamero, M., Blasco, M.A., and Serrano, M. (2009). The Ink4/Arf locus is a barrier for iPS cell reprogramming. Nature *460*, 1136–1139.

Loose, M., Swiers, G., and Patient, R. (2007). Transcriptional networks regulating hematopoietic cell fate decisions. Curr. Opin. Hematol. *14*, 307–314.

Loughran, S.J., Kruse, E.A., Hacking, D.F., de Graaf, C.A., Hyland, C.D., Willson, T.A., Henley, K.J., Ellis, S., Voss, A.K., Metcalf, D., et al. (2008). The transcription factor Erg is essential for definitive hematopoiesis and the function of adult hematopoietic stem cells. Nat. Immunol. *9*, 810–819.

McKinney-Freeman, S., Cahan, P., Li, H., Lacadie, S.A., Huang, H.-T., Curran, M., Loewer, S., Naveiras, O., Kathrein, K.L., Konantz, M., et al. (2012). The transcriptional landscape of hematopoietic stem cell ontogeny. Cell Stem Cell *11*, 701–714.

Mikkola, H.K., Fujiwara, Y., Schlaeger, T.M., Traver, D., and Orkin, S.H. (2003). Expression of CD41 marks the initiation of definitive hematopoiesis in the mouse embryo. Blood *101*, 508–516.

Mitjavila-Garcia, M.T., Cailleret, M., Godin, I., Nogueira, M.M., Cohen-Solal, K., Schiavon, V., Lecluse, Y., Le Pesteur, F., Lagrue, A.H., and Vainchenker, W. (2002). Expression of CD41 on hematopoietic progenitors derived from embryonic hematopoietic cells. Development *129*, 2003–2013.

Nam, Y.J., Song, K., Luo, X., Daniel, E., Lambeth, K., West, K., Hill, J.A., DiMaio, J.M., Baker, L.A., Bassel-Duby, R., and Olson, E.N. (2013). Reprogramming of human fibroblasts toward a cardiac fate. Proc. Natl. Acad. Sci. USA *110*, 5588–5593.

Ohnishi, K., Semi, K., Yamamoto, T., Shimizu, M., Tanaka, A., Mitsunaga, K., Okita, K., Osafune, K., Arioka, Y., Maeda, T., et al. (2014). Premature termination of reprogramming in vivo leads to cancer development through altered epigenetic regulation. Cell *156*, 663–677.

Okuda, T., van Deursen, J., Hiebert, S.W., Grosveld, G., and Downing, J.R. (1996). AML1, the target of multiple chromosomal translocations in human leukemia, is essential for normal fetal liver hematopoiesis. Cell *84*, 321–330.

Orkin, S.H., and Zon, L.I. (2008). Hematopoiesis: an evolving paradigm for stem cell biology. Cell *132*, 631–644.

Pereira, C.F., Chang, B., Qiu, J., Niu, X., Papatsenko, D., Hendry, C.E., Clark, N.R., Nomura-Kitabayashi, A., Kovacic, J.C., Ma'ayan, A., et al. (2013). Induction of a hemogenic program in mouse fibroblasts. Cell Stem Cell *13*, 205–218.

Pereira, C.F., Lemischka, I.R., and Moore, K. (2014). 'From blood to blood': de-differentiation of hematopoietic progenitors to stem cells. EMBO J. *33*, 1511–1513.

Pimanda, J.E., and Göttgens, B. (2010). Gene regulatory networks governing haematopoietic stem cell development and identity. Int. J. Dev. Biol. *54*, 1201–1211.

Pimanda, J.E., Ottersbach, K., Knezevic, K., Kinston, S., Chan, W.Y.I., Wilson, N.K., Landry, J.-R., Wood, A.D., Kolb-Kokocinski, A., Green, A.R., et al. (2007). Gata2, Fli1, and Scl form a recursively wired gene-regulatory circuit during early hematopoietic development. Proc. Natl. Acad. Sci. USA *104*, 17692–17697.

Ran, D., Shia, W.J., Lo, M.C., Fan, J.B., Knorr, D.A., Ferrell, P.I., Ye, Z., Yan, M., Cheng, L., Kaufman, D.S., and Zhang, D.E. (2013). RUNX1a enhances hematopoietic lineage commitment from human embryonic stem cells and inducible pluripotent stem cells. Blood *121*, 2882–2890.

Riddell, J., Gazit, R., Garrison, B.S., Guo, G., Saadatpour, A., Mandal, P.K., Ebina, W., Volchkov, P., Yuan, G.C., Orkin, S.H., and Rossi, D.J. (2014). Reprogramming committed murine blood cells to induced hematopoietic stem cells with defined factors. Cell *157*, 549–564.

Robb, L., Elwood, N.J., Elefanty, A.G., Köntgen, F., Li, R., Barnett, L.D., and Begley, C.G. (1996). The scl gene product is required for the generation of all hematopoietic lineages in the adult mouse. EMBO J. *15*, 4123–4129.

Robin, C., Ottersbach, K., Boisset, J.-C., Oziemlak, A., and Dzierzak, E. (2011). CD41 is developmentally regulated and differentially expressed on mouse hematopoietic stem cells. Blood *117*, 5088–5091.

Sandler, V.M., Lis, R., Liu, Y., Kedem, A., James, D., Elemento, O., Butler, J.M., Scandura, J.M., and Rafii, S. (2014). Reprogramming human endothelial cells to haematopoietic cells requires vascular induction. Nature *511*, 312–318.

Sekiya, S., and Suzuki, A. (2011). Direct conversion of mouse fibroblasts to hepatocyte-like cells by defined factors. Nature *475*, 390–393.

Sroczynska, P., Lancrin, C., Pearson, S., Kouskoff, V., and Lacaud, G. (2009). In vitro differentiation of mouse embryonic stem cells as a model of early hematopoietic development. Methods Mol. Biol. *538*, 317–334.

Sturgeon, C.M., Ditadi, A., Clarke, R.L., and Keller, G. (2013). Defining the path to hematopoietic stem cells. Nat. Biotechnol. *31*, 416–418.

Szabo, E., Rampalli, S., Risueño, R.M., Schnerch, A., Mitchell, R., Fiebig-Comyn, A., Levadoux-Martin, M., and Bhatia, M. (2010). Direct conversion of human fibroblasts to multilineage blood progenitors. Nature *468*, 521–526.

Takahashi, K., and Yamanaka, S. (2006). Induction of pluripotent stem cells from mouse embryonic and adult fibroblast cultures by defined factors. Cell *126*, 663–676.

Takahashi, K., Tanabe, K., Ohnuki, M., Narita, M., Ichisaka, T., Tomoda, K., and Yamanaka, S. (2007). Induction of pluripotent stem cells from adult human fibroblasts by defined factors. Cell *131*, 861–872.

Taoudi, S., Bee, T., Hilton, A., Knezevic, K., Scott, J., Willson, T.A., Collin, C., Thomas, T., Voss, A.K., Kile, B.T., et al. (2011). ERG dependence distinguishes developmental control of hematopoietic stem cell maintenance from hematopoietic specification. Genes Dev. *25*, 251–262.

Tsai, F.Y., Keller, G., Kuo, F.C., Weiss, M., Chen, J., Rosenblatt, M., Alt, F.W., and Orkin, S.H. (1994). An early haematopoietic defect in mice lacking the transcription factor GATA-2. Nature *371*, 221–226.

Vierbuchen, T., Ostermeier, A., Pang, Z.P., Kokubu, Y., Südhof, T.C., and Wernig, M. (2010). Direct conversion of fibroblasts to functional neurons by defined factors. Nature *463*, 1035–1041.

Wilson, N.K., Foster, S.D., Wang, X., Knezevic, K., Schütte, J., Kaimakis, P., Chilarska, P.M., Kinston, S., Ouwehand, W.H., Dzierzak, E., et al. (2010). Combinatorial transcriptional control in blood stem/progenitor cells: genome-wide analysis of ten major transcriptional regulators. Cell Stem Cell *7*, 532–544.

Zhao, Z., Zuber, J., Diaz-Flores, E., Lintault, L., Kogan, S.C., Shannon, K., and Lowe, S.W. (2010). p53 loss promotes acute myeloid leukemia by enabling aberrant self-renewal. Genes Dev. *24*, 1389–1402.

Zovein, A.C., Hofmann, J.J., Lynch, M., French, W.J., Turlo, K.A., Yang, Y., Becker, M.S., Zanetta, L., Dejana, E., Gasson, J.C., et al. (2008). Fate tracing reveals the endothelial origin of hematopoietic stem cells. Cell Stem Cell *3*, 625–636.

Cell Reports, Volume *9*

Supplemental Information

Direct Reprogramming of Murine Fibroblasts to Hematopoietic Progenitor Cells

Kiran Batta, Magdalena Florkowska, Valerie Kouskoff, and Georges Lacaud

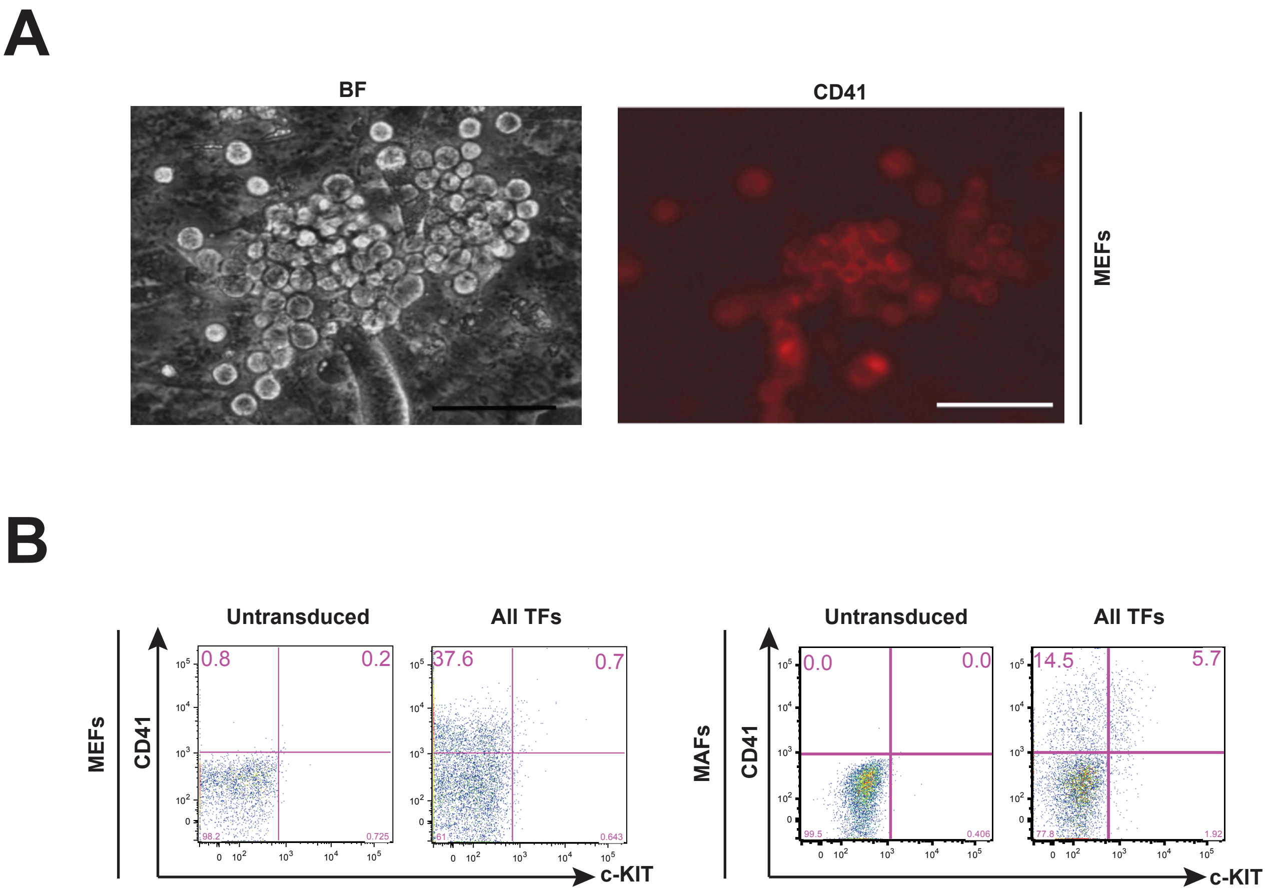

Figure S1 (related to Figure 1)

(A) Immunostaining of transduced MEFs at day 12 with a CD41-PE antibody. No staining was observed in untransduced MEFs. (B) FACS analyses for hematopoietic cell surface markers on all factor transduced MEFs and MAFs at day 21.

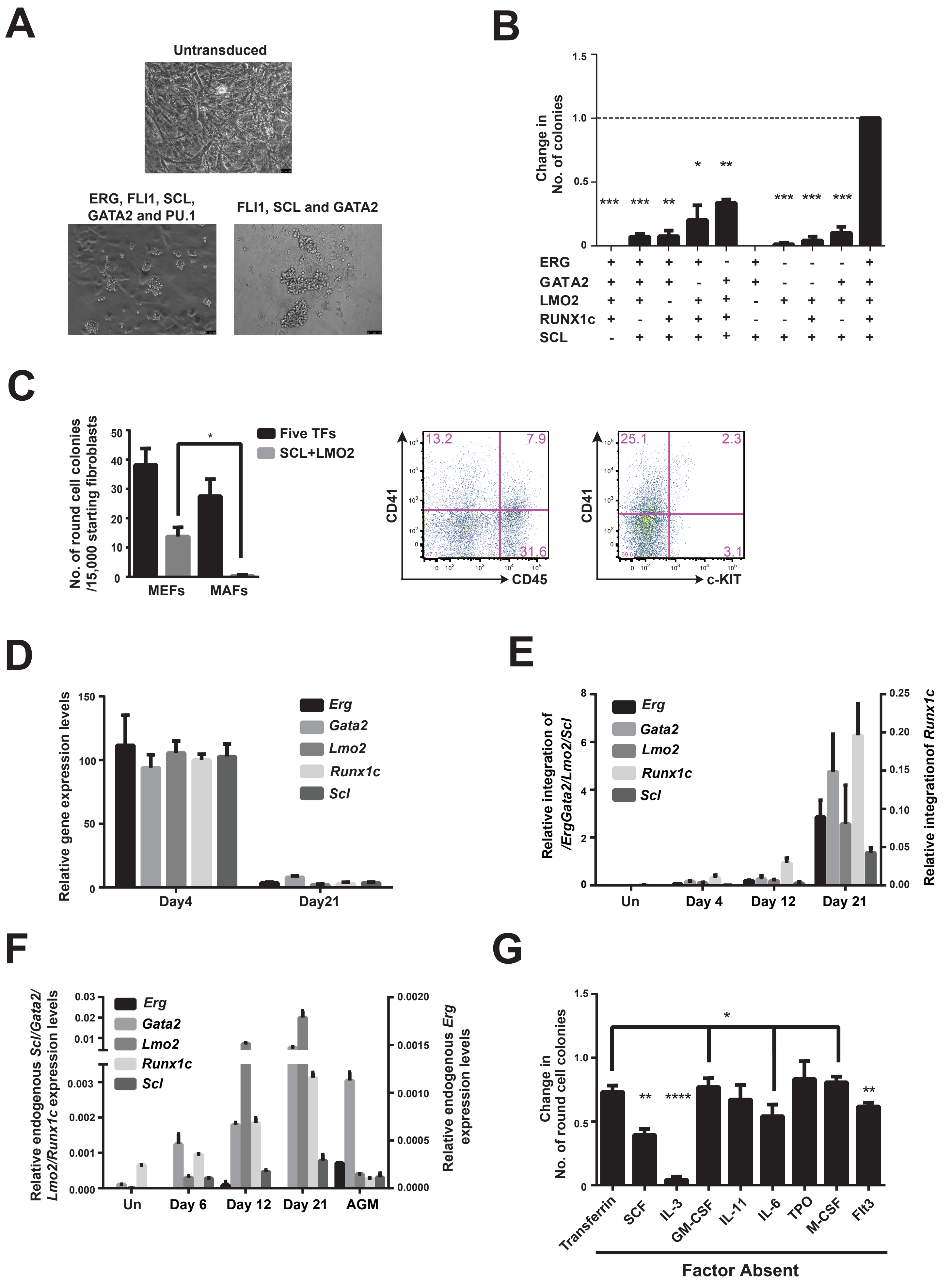
A
Untransduced
ERG, FLI1, SCL, GATA2 and PU.1
FLI1, SCL and GATA2
B
Change in No. of colonies
ERG
GATA2
LMO2
RUNX1c
SCL
C
No. of round cell colonies /15,000 starting fibroblasts
Five TFs
SCL+LMO2
MEFs
MAFs
CD41
CD45
c-KIT
D
Relative gene expression levels
Erg
Gata2
Lmo2
Runx1c
Scl
Day4
Day21
E
Relative integration of /ErgGata2/Lmo2/Scl
Relative integrationof Runx1c
Un
Day 4
Day 12
Day 21
F
Relative endogenous Scl/Gata2/ Lmo2/Runx1c expression levels
Relative endogenous Erg expression levels
Un
Day 6
Day 12
Day 21
AGM
G
Change in No. of round cell colonies
Transferrin
SCF
IL-3
GM-CSF
IL-11
IL-6
TPO
M-CSF
Flt3
Factor Absent

Figure S2 (related to Figure 2)

(A) Bright-field images of round cell colonies generated by transduced MEFs with the indicated TFs at day 18.

(B) Relative change in the number of round cell colonies visible at day 15 following transduction of MAFs with the indicated combination of TFs. The number of colonies generated by the complete five TFs combination is attributed a value of 1. Mean ± SEM of three independent experiments is shown (n=3) (C) Number of round cell colonies generated by 15K MEFs/MAFs transduced with either five TFs or only SCL and LMO2 (left). Mean ± SEM of two independent experiments is shown (n=2). FACS analysis of day 21 SCL and LMO2 transduced MEFs cultures (right). (D) Relative expression levels with respect to β-actin of exogenously introduced TFs in day 21 transduced cells and day 4 transduced cells (n=3, mean ± SD). (E) Relative integration of vectors encoding the different TFs in untransduced (Un) and transduced MEFs at day 4, day 12 and day 21 in respect to the endogenous *PARP* gene (n=3, mean ± SD). (F) Relative expression levels of endogenous genes with respect to β-*actin* in untransduced (Un) MEFs, day 6, day 12 and day 21 transduced MEFs and control E10.5 AGM cells. Mean ± SD of triplicates of a single experiment is shown. (G) Change in the number of round cell colonies generated after five TF transduction in media depleted of the indicated cytokines or growth factors. Mean ± SEM of four independent experiments is shown (n=4). Asterisk(s) indicates significant differences (Student t-test, *p <0.05, **p<0.01, ***p<0.0005, ****p<0.0001).

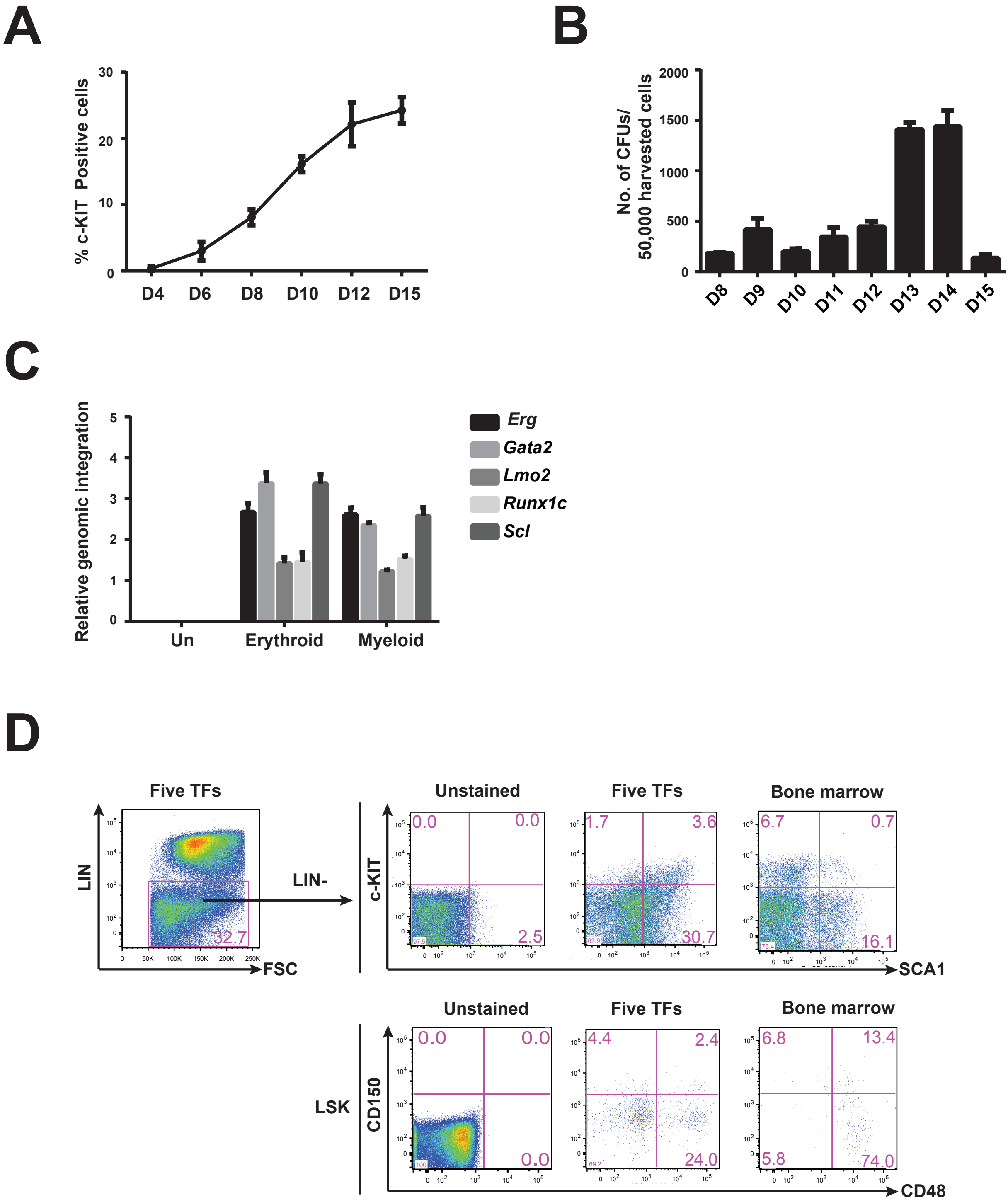

Figure S3 (related to Figure 3)

(A) FACS analyses of the emergence of c-KIT+ cells in cultures of transduced MAFs at the indicated days. Mean ± SEM of three independent experiments is shown (n=3) (B) CFU potential of five TF transduced MAFs at the indicated days of cultures. Bars represent mean ± SEM of triplicates of a representative experiment (n=3) (C) Relative genomic integration of vectors for all five TFs in untransduced (Un) and reprogrammed MEFs in respect to the endogenous *PARP* gene. MEFs transduced with five TFs were cultured on OP9. On day 14 cells were sorted into erythroid (TER119+) or myeloid (CD11b+) fractions and subjected to genomic DNA extractions and subsequent qRT-PCR. Data presented are mean ± SD of a representative experiment performed in triplicates (n=2). (D) FACS analyses using the indicated markers of phenotypic HSCs in reprogrammed MEFs expanded on OP9-DL1 for two weeks and control bone marrow.

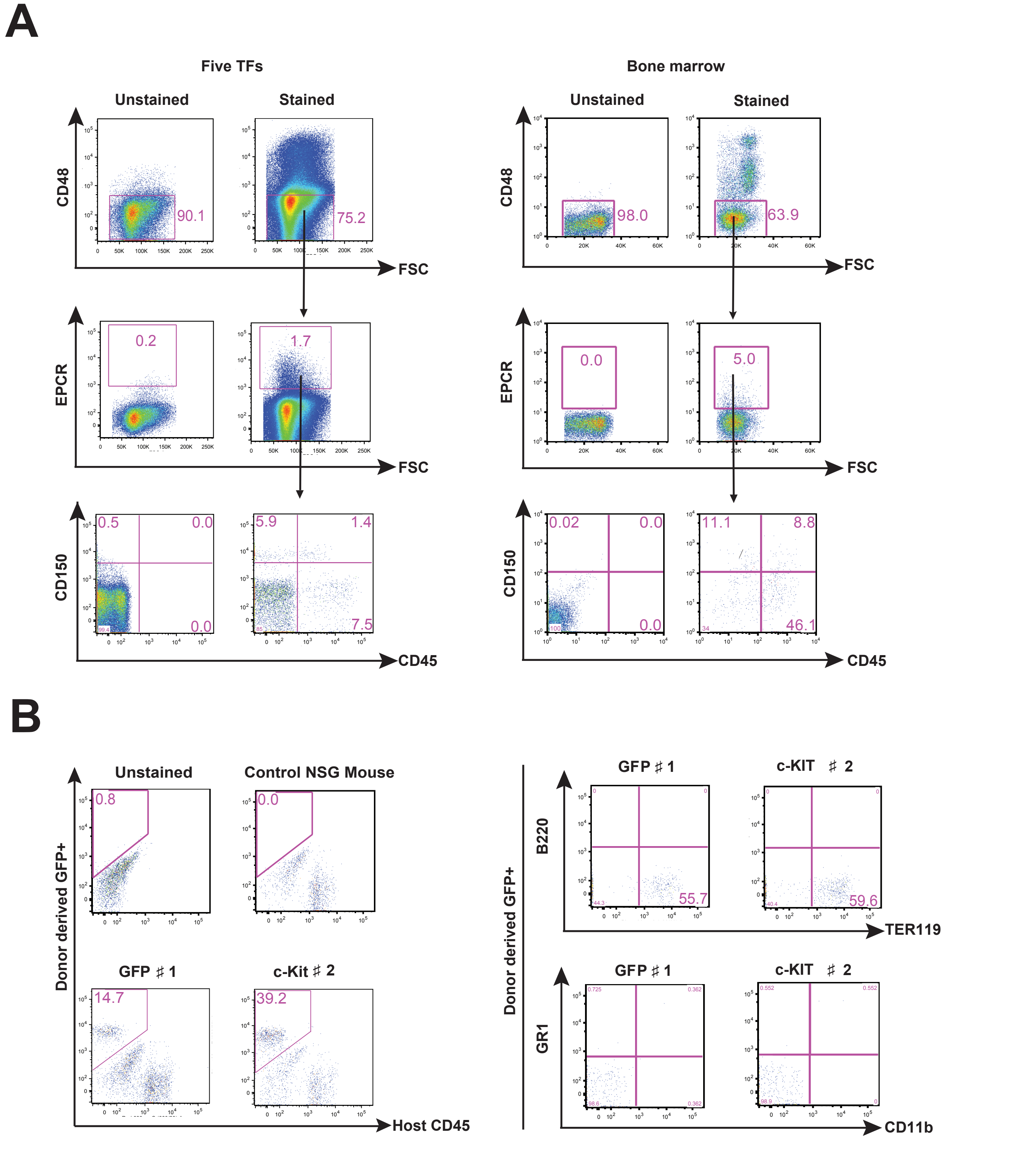

Figure S4 (related to Figure 3)

(A) FACS analyses using the indicated markers of phenotypic HSCs in reprogrammed MEFs expanded on OP9-DL1 for two weeks and control bone marrow. (B) Representative FACS analyses of peripheral blood of mice 2 weeks after transplantation for donor derived GFP positive cells and host CD45 (right). FACS analyses of donor derived cells (GFP gated) for the hematopoietic cell surface markers B220, TER119, GR1 and CD11b (left).

A

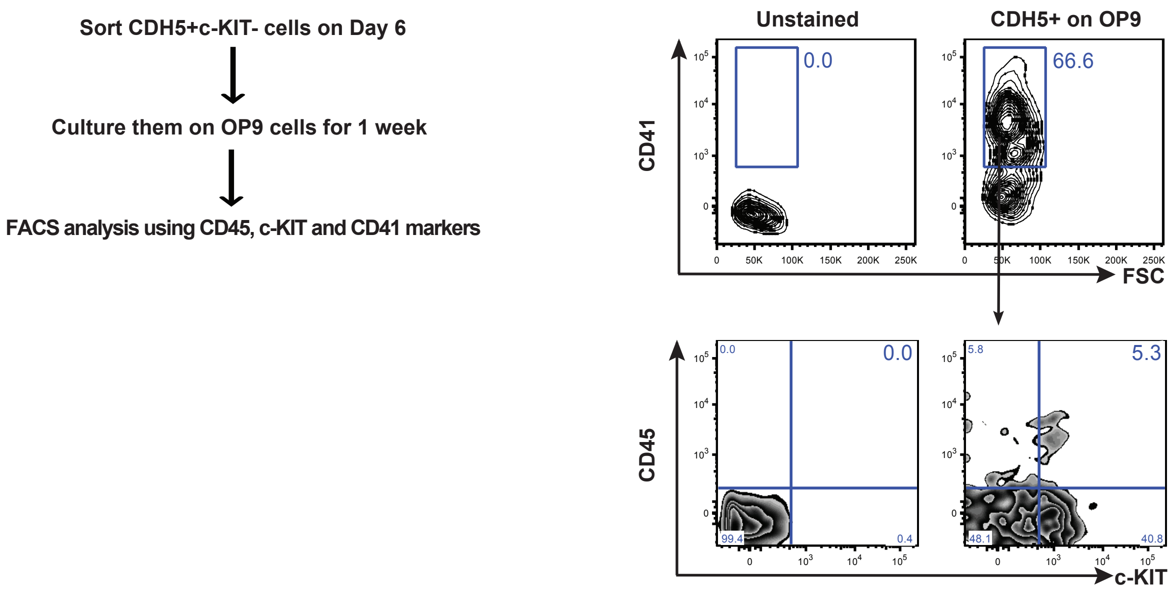

Figure S5 (related to Figure 5)

(A) CDH5+ c-KIT- cells were sorted from transduced day 6 MEFs and cultured on OP9 stromal layers for 7 days. FACS analyses were performed for the indicated markers (n=2).

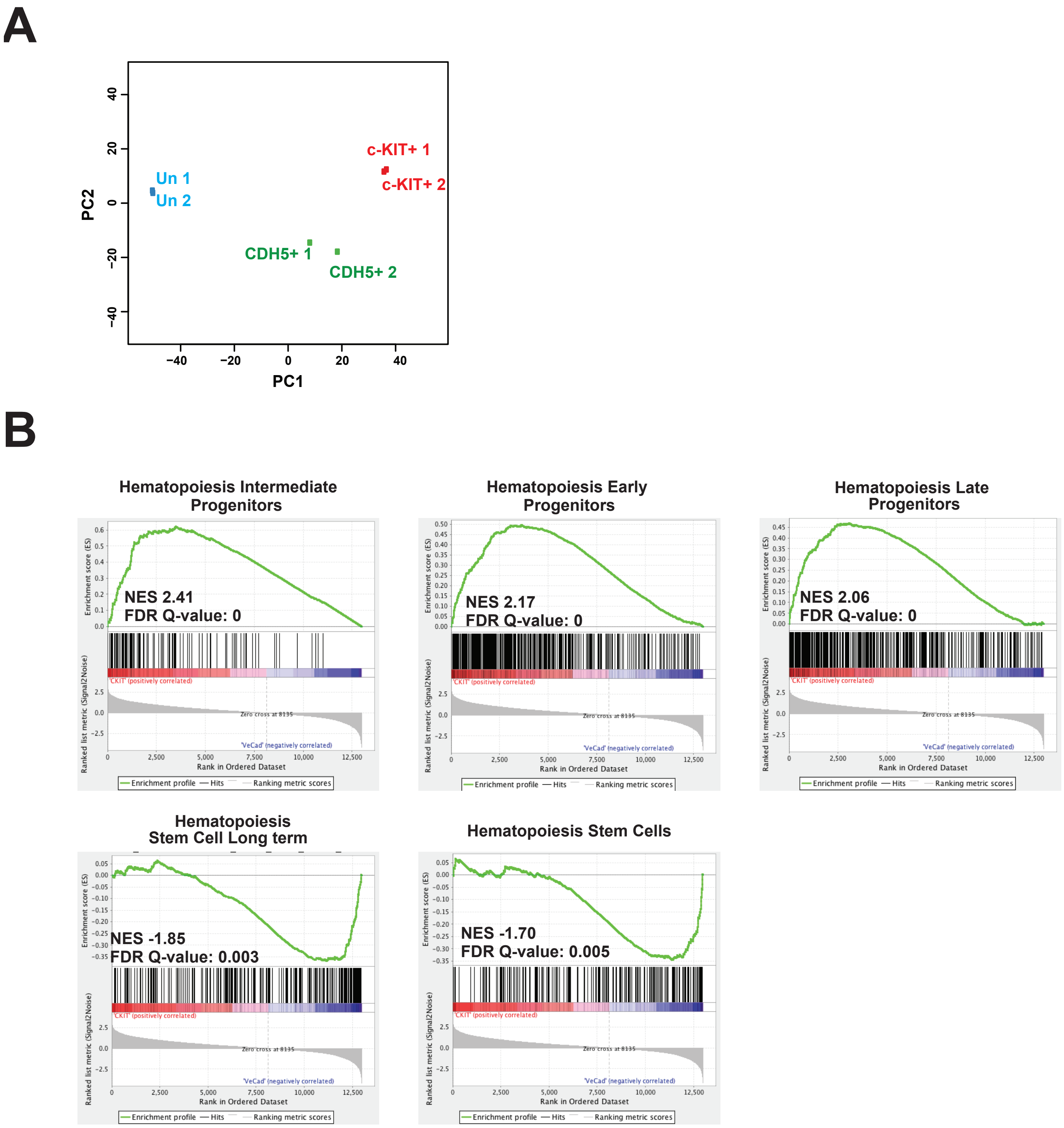

Figure S6 (realted to Figure 6)

(A) PCA analyses of DEGs among untreated (Un), CDH5+ and c-KIT+ positive cells. (B) GSEA analyses with the indicated gene data sets (Ivanova, 2002) comparing CDH5+ and c-KIT+ transcriptomes.

Table S1: (related to Figure 1)
List of the 19 TFs screened for reprogramming to blood

Gene Name	Gene ID
CEBP-α	NM_007678.3
CEBP-β	NM_009883.3
c-Fos	NM_010234.2
Erg	NM_133659.2
Ets1	NM_001038642.1
Etv2	NM_007959.2
Etv6	NM_007961.3
Fli1	NM_008026.4
GATA1	NM_008089.1
GATA2	NM_008090.4
Gfi1	NM_010278.2
Gfi1b	NM_008114.2
HoxB4	NM_010459.6
Lmo2	NM_008505.3
PU.1	NM_011355.1
Runx1b	NM_001111022.2
Runx1c	NM_001111023.2
Scl	NM_011527.2
Sox7	NM_011446.1

Table S2 : (related to Figure 6)

Gene ontology analysis of significantly enriched biological processes in the 3 clusters of genes differentially expressed among untreated fibroblasts, day 8 CDH5+ and day 12 c-Kit positive cells.

Cluster 1	Cluster 2	Cluster 3
Cell adhesion	Cell adhesion	Immune response
Biological adhesion	Biological adhesion	Defense response
Heart development	Angiogenesis	Leukocyte migration
Actin filament-based process	Blood vessel development	Cell activation
Extracellular matrix organization	Vasculature development	Response to wounding
Extracellular structure organization	Regulation of cell migration	Taxis
Enzyme linked receptor protein signalling pathway	Blood vessel morphogenesis	Leukocyte activation
Skeletal system development	Regulation of cell motion	Response to bacterium
Actin cytoskeleton organization	Regulation of locomotion	Mast cell activation

Movie S1: related to Figure 2

Time lapse imaging of 5-TFs transduced MEFs from day 3 to 13 of reprogramming.

Supplemental Experimental Procedures

TF cloning and lentiviruses production

Hematopoietic TFs cDNAs were cloned with a N-terminal FLAG tag in lentiviral vectors under the EF1 promoter. Viral particles were produced in HEK293T cells with third generation lentiviral packaging plasmids. Western blotting with FLAG antibodies was performed to quantitate and normalize the titers of viruses.

Flow cytometry

The following antibodies were used: CD45.1 APC (A20), CD45-PerCP Cy5.5 (30-F11;Biolegend), CD150-PerCP Cy5.5 (TC15-12F12.2), SCA1-PE-Cy7 (D7), CD201-APC (1560), CD48-PE (OX-78), CD48-APC (HM48-1), CD41-PE-Cy7 (MWReg30), c-KIT-APCeFluor780 (2B8), CDH5–APC (eBioBV13), TER119- PE-Cy7 (TER-119), CD11b-APC (M1/70), GR1-PE-Cy7 (RB6-8C5), CD19-PE-Cy7 (eBio1D3), B220-PE (RA3-6B2), CD25-APC (PC61.5), CD71-bio (R17217), CD31-Bio (Mec 13-3), CD41-Bio (eBio-MWReg30), CD45-Bio and PE-Cy7 (30-F11), c-KIT-Bio (2B8), Strep-BV421, Strep-PECy7 and mouse hematopoietic lineage biotin panel (all from eBioscience). Acquisitions were performed on LSRII (BD Biosciences) and data were analyzed using FlowJo software. Cell sortings were performed on AriaII or AriaIII sorters (BD Biosciences).

Lineage specific cultures

Multilineage potential of sorted c-KIT positive cells was tested by culturing them in (1X IMDM supplemented with plasma-derived serum (PDS;Antech), 10% protein-free hybridoma medium (PFM; Gibco), 0.5 mM Ascorbic Acid, 4.5x10-4 M MTG, 2 mM L-glutamine, 80 mg/ml transferrin and 50 µg/ml penicillin-streptomycin in addition to the following specific cytokines/growth factors; 1% IL3, 1% of GM-CSF and 10 ng/ml M-CSF for myeloid, 1% IL3, 1% c-KIT ligand and 4 U/ml Erythropoietin (Ortho-Biotech) for erythroid and 1% Thrombopoietin, 10 ng/ml IL6 and 5 ng/ml IL11 (R&D Systems) for megakaryocytes. Lymphoid cultures were carried out in the presence of 1% c-KIT ligand, 5 ng/ml Flt3 ligand and 1 ng/ml IL7 (B-lymphocytes) or 0.25 ng/ml (T-lymphocytes). To test presence of immunophenotypic HSCs day 12 c-KIT sorted five factor reprogrammed cells were cultured in medium supplemented with 1% c-KIT ligand, 1% IL3, 100 ng/ml Flt3 ligand and 10 ng/ml IL6 for two weeks.

Hematopoietic CFU-C assay

In order to evaluate the progenitor potential of the cells subjected to reprogramming, 50,000 cells were re-plated in triplicate in semi-solid media containing 55% methylcellulose (VWR) and supplemented with the cytokines used in reprogramming medium (see Viral transduction and culture). The cultures were maintained in

$37^0C/5\% CO_2$ atmosphere and scored for the presence of hematopoietic colonies after 10-12 days.

Phagocytosis assay and cytospin analysis

Active phagocytosis was tested by adding 10 µl of fluorescent red conjugated carboxylate-modified polystyrene beads (0.5 um diameter, Sigma) to 100 µl aliquots of day 21 $CD45^+CD11b^+$ sorted reprogrammed cells ($3x10^6$/ml) and subsequent incubation for 2 hours at $37^0C/5\% CO_2$ atmosphere with occasional shaking. Cells were washed twice with 10% FCS in PBS to remove any beads that were not phagocytised and subjected to standard cytospin procedure.
For cytospin analysis 30,000 cells suspended in 150 µl of medium were spun at 800 rpm for 5 minutes with low acceleration onto a glass slide. After air-drying cells were fixed in methanol for 10 minutes, washed and stained with May-Grunwald dye for 3 minutes. Following 5 rounds of washing, slides were submerged into Giemsa stain for 20 minutes, rinsed with water and air-dried. Subsequently mounted slides were scanned using Leica Scan Software.

Acetylcholinesterase staining

Acetycholinesterase staining was performed on cells generated from day 12 c-KIT^+ sorted reprogrammed $p53^{-/-}$ MEFs or whole bone marrow cells after culture in megakaryocytic medium. 30,000 cells were span onto glass slide at 800 rpm for 5 minutes and air dried. The cells were next fixed in ice-cold acetone for 5 minutes and air-dried. Samples were then covered with a staining solution of 0.5 mg/ml acetylthiocholine iodide, 5mM sodium citrate, 3mM copper(II) sulphate, 0.5mM potassium ferricyanide in 0.1M sodium phosphate buffer (pH6). Incubation was carried out for 3-6 hours in a humid chamber in the dark. The cells were then fixed in 95% EtOH for 10 minutes, rinsed with water and air dried. Counterstain in Harris' haematoxylin solution was carried out for 30 seconds immediately followed by brief destain in acid alcohol (1% HCl in 70% EtOH). Finally slides were rinsed with water and left to air dry. Images were captured on a Leica DMI3000B microscope.

Genomic PCR

Genomic DNA was isolated by lysis followed by proteinase K digestion. The integration of exogenous transcription factors was determined by PCR with SYBR green master mix and primers specific to FLAG tag and individual TF. For the detection of BCR and TCR gene rearrangements, $p53^{-/-}$ reprogrammed MEFs, cultured on OP9/OP9-DL1, were sorted for B220 and CD19 (B-lymphocytes) or CD25 (T-lymphocytes) respectively. Genomic DNA was isolated from 100.000 cells. D_H to J_H1-2 and D_H to J_H3 gene rearrangements were detected by nested PCR as reported earlier (Schlissel et al., 1991). TCR-β chain gene rearrangements were detected by a single round of PCR with the following primers: 5'-GCACCTGTGGGGAAGAAACT-3'; 5'-TGAGAGCTGTCTCCTACTATCGATT-3'. Products of both experiments were subjected to electrophoresis and the bands excised from the gel and sequenced.

Quantitative RT-PCR analysis

Total RNA was isolated using QIAGEN RNA Prep KIT (Qiagen) and cDNAs were generated with Omniscript RT kit (Qiagen). The QRT-PCR reactions were prepared using TaqMan Universal PCR Master Mix (Applied Biosystems) and universal probe library. The expression levels of endogenous TFs were determined with primers specific to the 3'UTR of their transcripts.

Immunofluorescence

For CDH5 staining, five TFs transduced cells cultured on gelatine-coated coverslips were washed with PBS and fixed with 0.4% formaldehyde (Sigma) in PBS for 20 minutes at RT. Cells were then permeabilized with a 0.2% TritonX (Sigma) solution in PBS before to be blocked with 10% goat serum (Gibco) for 10 minutes at RT. The cells were then incubated with a CDH5-biotin primary antibody (eBioBV14, eBioscience) at 1:100 dilution overnight at 4C and the next day with a secondary anti-rat Alexa647 antibody (Invitrogen) in 1:400 dilution for 1 hour. After washes, the cells were mounted with ProLong® Gold antifade reagent with DAPI (Invitrogen) and left at 4^{0}C overnight. Imaging was performed using a Zeiss Axiovert 200M microscope and a Andor iXon DU888+ camera. The system utilises Metamorph (Molecular Devices) software. Subsequent analysis was performed in Adobe Photsohop and ImageJ programmes.

Supplemental references

Ivanova, N.B. (2002). A stem cell molecular signature. Science *298*, 601–604.

Schlissel, M., Voronova, A., and Baltimore, D. (1991). Helix-loop-helix transcription factor E47 activates germ-line immunoglobulin heavy-chain gene transcription and rearrangement in a pre-T-cell line. Genes Dev *5*, 1367–1376.
